# Cell Proliferation, Migration, and Neurogenesis in the Adult Brain of the Pulse Type Weakly Electric Fish, *Gymnotus omarorum*

**DOI:** 10.3389/fnins.2017.00437

**Published:** 2017-08-17

**Authors:** Valentina Olivera-Pasilio, Moira Lasserre, María E. Castelló

**Affiliations:** ^1^Desarrollo y Evolución Neural, Departamento de Neurociencias Integrativas y Computacionales, Instituto de Investigaciones Biológicas Clemente Estable, Ministerio de Educación y Cultura Montevideo, Uruguay; ^2^Departamento de Histología y Embriología, Facultad de Medicina, Universidad de la República Montevideo, Uruguay; ^3^IIBE “Histología de Sistemas Sensoriales”, Unidad Asociada F. de Medicina Montevideo, Uruguay

**Keywords:** cerebellum, olfactory bulb, tectum opticum, CldU, doublecortin, HuC/HuD, tyrosine hydroxylase, retrograde tracing

## Abstract

Adult neurogenesis, an essential mechanism of brain plasticity, enables brain development along postnatal life, constant addition of new neurons, neuronal turnover, and/or regeneration. It is amply distributed but negatively modulated during development and along evolution. Widespread cell proliferation, high neurogenic, and regenerative capacities are considered characteristics of teleost brains during adulthood. These anamniotes are promising models to depict factors that modulate cell proliferation, migration, and neurogenesis, and might be intervened to promote brain plasticity in mammals. Nevertheless, the migration path of derived cells to their final destination was not studied in various teleosts, including most weakly electric fish. In this group adult brain morphology is attributed to sensory specialization, involving the concerted evolution of peripheral electroreceptors and electric organs, encompassed by the evolution of neural networks involved in electrosensory information processing. In wave type gymnotids adult brain morphology is proposed to result from lifelong region specific cell proliferation and neurogenesis. Consistently, pulse type weakly electric gymnotids and mormyrids show widespread distribution of proliferation zones that persists in adulthood, but their neurogenic potential is still unknown. Here we studied the migration process and differentiation of newborn cells into the neuronal phenotype in the pulse type gymnotid *Gymnotus omarorum*. Pulse labeling of S-phase cells with 5-Chloro-2′-deoxyuridine thymidine followed by 1 to 180 day survivals evidenced long distance migration of newborn cells from the rostralmost telencephalic ventricle to the olfactory bulb, and between layers of all cerebellar divisions. Shorter migration appeared in the tectum opticum and torus semicircularis. In many brain regions, derived cells expressed early neuronal markers doublecortin (chase: 1–30 days) and HuC/HuD (chase: 7–180 days). Some newborn cells expressed the mature neuronal marker tyrosine hydroxylase in the subpallium (chase: 90 days) and olfactory bulb (chase: 180 days), indicating the acquisition of a mature neuronal phenotype. Long term CldU labeled newborn cells of the granular layer of the corpus cerebelli were also retrogradely labeled “*in vivo*,” suggesting their insertion into the neural networks. These findings evidence the neurogenic capacity of telencephalic, mesencephalic, and rhombencephalic brain proliferation zones in *G. omarorum*, supporting the phylogenetic conserved feature of adult neurogenesis and its functional significance.

## Introduction

Neurogenesis is the main mechanism of adult brain plasticity that enables the continuation of brain development, the constant addition of new neurons and/or the neuronal turnover (Barker et al., 2011; Alunni and Bally-Cuif, 2016). It has been demonstrated in a wide range of animals, from cnidarians to mammals, including humans (Altman, 1963, 1969; Altman and Das, 1965; Sullivan et al., 2007; Galliot and Quiquand, 2011). Neurogenesis is progressively restricted during animal development and negatively modulated along evolution. Its spatial distribution remains widespread in all brain divisions of adult anamniotes, particularly in teleost fish (Cayre et al., 2002; Lindsey and Tropepe, 2006; Kaslin et al., 2008; Barker et al., 2011; Grandel and Brand, 2013). However, it is almost confined to two zones in the telencephalon of adult mammals (Ma et al., 2008; Altman, 2011; Vadodaria and Gage, 2014).

Soon after the discovery of adult neurogenesis in mammals by Altman (Altman, 1962, 1963; Altman and Das, 1965, 1966) the widespread distribution of cell proliferation in the brain of adult teleost was evidenced by tritiated thymidine (3H-thymidine) labeling (*Brachydanio rerio*: Rahmann, 1968; *Lebistes reticulatus*: Richter and Kranz, 1970). In spite of ultrastructural evidences of adult neurogenesis in mammals (Kaplan and Hinds, 1977), the field of cell proliferation and neurogenesis in both amniotes and anamniotes resumed after a 20 years hindrance (Gross, 2000). Even though the spatial distribution of proliferation zones was since then evidenced in several teleost, numerous taxa remain underexplored. One of the earliest and most thoroughly studied anamniotes is the wave type weakly electric gymnotid *Apteronotus leptorhynchus*, which is considered a classical biological model in the field. Weakly electric fish are also good models for the study of brain evolution sub-serving variations in animal behavior (Albert et al., 1998). The peculiar adult brain morphology of weakly electric fish is associated to the relevance of the electrosensory modality for these fish lifestyle (Evans, 1940; Bennett, 1971; Hodos and Butler, 1997; Kotrschal et al., 1998; Meek and Nieuwenhuys, 1998; Ito et al., 2007; Shumway, 2008). It results from the differential growth of portions of the neural tube that progressively differentiate into brain vesicles followed by the subsequent formation and differential growth of brain structures derived from the alar plate, as shown in wave (Leyhausen et al., 1987; Lannoo et al., 1990) and pulse (Iribarne and Castelló, 2014) gymnotids, and pulse mormyrids (Haugedé-Carré et al., 1977, 1979; Radmilovich et al., 2016). The maintenance of adult brain morphology as fish body grows indefinitely depends in turn on heterogeneous cell proliferation and neurogenesis. Thus, neurogenesis can be considered as a plastic mechanism to maintain the “proper mass” of neural tissue controlling particular functions (Jerisson, 1973) or for the “matching” between peripheral elements and brain processing power (Zupanc, 2006, 2008, 2011), contributing to the functional specialization (Grandel et al., 2006). Brain proliferation zones also persist in adulthood in *G. omarorum* (Olivera-Pasilio et al., 2014) and *Brachyhypopomus gauderio* (Dunlap et al., 2011) showing overall similarities with their distribution in *A. leptorhynchus* (Zupanc and Horschke, 1995; Zupanc, 2006, 2008).

Adult *G. omarorum* newborn cells appear to differ in their pace of migration (Olivera-Pasilio et al., 2014). Newborn cells show long distance displacement at the rostral part of the telencephalon, suggesting a migration process similar to what occurs in amniotes (Altman, 1969; Lim et al., 2008) including humans (Wang et al., 2011) and birds (Goldman and Nottebohm, 1983; Nottebohm, 2002; Barnea and Pravosudov, 2011), and anamniotes (*Danio rerio*: Adolf et al., 2006; Kishimoto et al., 2011). Other brain region of comparative interest are the cerebellum (Cb), the dorsal, ventral, and posterior subdivisions of the dorsolateral zone of the dorsal telencephalon (considered homologous of the amniote hippocampus; Zupanc, 2006) and the tectum opticum (TeO). Newborn cells originated from these proliferation zones also display long range and/or relatively fast migration, as evidenced by the almost complete displacement of derived cells between cerebellar layers after a 30 days chase (Olivera-Pasilio et al., 2014). The location, as well as shape and appearance of the nuclei suggest that newborn cells are in the process of differentiation as shown in other teleost (Zupanc et al., 1996, 2005; Kaslin et al., 2009; Delgado and Schmachtenberg, 2011; Teles et al., 2012).

In this manuscript, we further analyze in adult *G. omarorum* the migration process of derived cells by pursuing their location from the proliferation zones to their final destination. We also studied the differentiation of newborn cells into the neuronal phenotype by demonstrating the co-localization of long-term thymidine analog labeling and expression of early neuronal markers doublecortin (DCX) and HuC/HuD or the mature neuronal marker tyrosine hydroxylase (TH). We found evidences of neurogenesis in several brain regions of the telencephalon, mesencephalon, and rhombencephalon. We further evidenced the insertion of newborn cells into neural circuits by the demonstration of long term thymidine analog labeling and “*in vivo*” retrogradely labeling of cerebellar granular cells. These findings contribute to support the widespread distribution of brain proliferation zones and their neurogenic capacity in teleost.

## Materials and methods

### General procedures

#### Animals

Thirteen adult specimens of *G. omarorum* (Richer-de-Forges et al., 2009; 8 males, 2 females and 3 of non-determined sex; weight: 6.26 ± 2.94; total fish length: 12.23 ± 2.04 mean ± SD) were collected from Laguna del Cisne, Uruguay (latitude 35°50 S, longitude 55°08 W). According to the measured length of the specimens, which is about half of the maximal length (Richer-de-Forges et al., 2009), we estimate that most of the fish used in this study have already reached the adult period, corresponding to the first gonadal maturation, at the age of 1 year (Balon, 1975; Barbieri and Cruz, 1983).

Macroscopic gonadal morphology was confirmed in 10 of the specimens studied. Animals were kept in individual tanks on a 12 h:12 h light: dark cycle and daily fed with *Tubifex tubifex*. Water conductivity was adjusted to 200 μs and temperature maintained at 20–25°C.

#### Anesthesia

All procedures were performed in accordance to the guidelines of CHEA (Comisión Honoraria de Experimentación Animal, ordinance number: 4332-99, Universidad de la República). Experiments were approved by the Animal Ethics Committee of the Instituto de Investigaciones Biológicas Clemente Estable (protocol number 010/09/2011).

For the application of Neurobiotin tracer (Vector Laboratories, Burlingame, CA, USA) in the corpus cerebelli (CCb), animals were anesthetized by immersion in 3-aminobenzoic acid ethyl ester (MS-222, Sigma-Aldrich, St Louis, MO, USA, at a dose of 120 mg/l) maintained by gill perfusion of the same anesthetic solution during surgery.

For transcardial fixation, fish were deeply anesthetized by immersion in MS-222 (500 mg/l, Sigma-Aldrich) followed by gill perfusion of the same anesthetic solution.

#### Fixation and tissue sectioning

Animals were first perfused with 20–30 ml of saline (0.7% NaCl solution) in order to wash blood out from the circulatory system, followed by 10% paraformaldehyde (Sigma-Aldrich) dissolved in phosphate buffer 0.1 M, pH 7.4 (PB; Carlo Erba, Val de Reuil, France). Brains were dissected out and post fixed in the same fixative solution for 24 h at 4°C. After embedding the brains in a gelatin/albumin (Sigma-Aldrich) mixture denatured with glutaraldehyde (Sigma-Aldrich), frontal serial sections (60 μm) were obtained with a vibratome (Leica VT1000S, Wetzlar, Germany) and serially collected in 24 or 48 multiwell plates.

**Figure 1:**
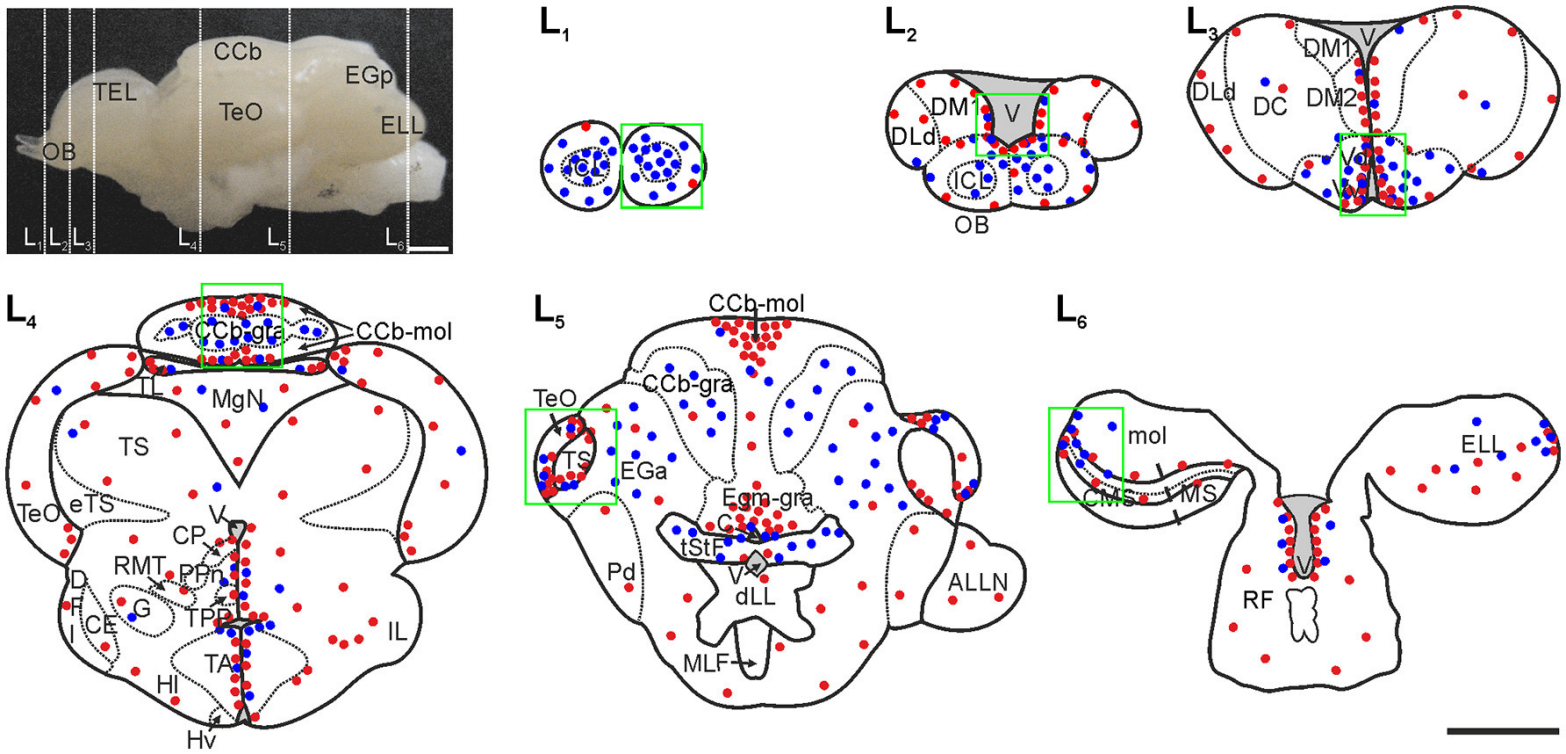
Spatial-temporal distribution of brain labeled cells in adult *G. omarorum* after short (1 day) and long (30 days) survivals following CldU administration. Labeled cells are qualitatively represented by red and blue dots (after chases of 1 day and 30 days, respectively) in the schematic diagrams of frontal sections (L1–L6) corresponding to the planes indicated by the dotted lines over the lateral view of the brain. Regions of interest of this work are indicated by the green squares. Modified from Olivera-Pasilio et al. (2014). CCb-mol, Cerebellum, molecular layer; CCb, Corpus cerebelli; CP, Central-posterior nucleus; CMS, Centromedial segment; DFl, Nucleus diffusus lateralis of the inferior lobe; EGa, EGa-gra, Eminentia granularis pars anterior, granular layer; ELL, Electrosensory lateral line lobe; eTS, Torus semicircularis efferents; Hv, Hypothalamus ventralis; ICL, Internal cell layer; IL, Inferior lobe; DM1, Dorsomedial telencephalon; DLd, Dorsolateral telencephalon, dorsal subdivision; DM1, Dorsomedial telencephalon, subdivision 1; DM2, Dorsomedial telencephalon, subdivision 2; MgN, Magnocellular mesencephalic nucleus; MS, Medial segment; mol, Molecular layer (ELL); PPn, Prepacemaker nucleus; RF, Reticular formation; TA, Nucleus tuberis anterior; TEL, Telencephalon; TeO, Tectum Opticum; TL, Torus longitudinalis; TPP, Periventricular nucleus of the posterior tuberculum; tStF, Tractus stratum fibrosum; V, Ventricle; Vd, Ventral telencephalon, dorsal subdivision; Vv, Ventral telencephalon, ventral subdivision. Scale bars: 1 mm.

In many cases, sections from most brain regions were processed in parallel, though we focused on certain rostral-caudal levels. At these levels, long distance and/or high pace of migration of newborn cells were previously evidenced (Olivera-Pasilio et al., 2014): the medial (L1) and caudal region (L2) of OB, corresponding to plates 1 of *Gymnotus carapo* atlas (Corrêa et al., 1998) and plate 38 of *A. lepthorhynchyus* atlas (Maler et al., 1991); the rostral region of the subpallium (L3) that corresponds to plates 2 and 32 of the mentioned atlases, respectively; the medial region and caudal pole of the midbrain TeO and torus semicircularis (TS; L4, and L5), the rostral pole of the rhombencephalic corpus cerebelli (CCb; L5), and the caudal pole of the rhombencephalic electrosensory lateral line lobe (ELL; L6; Figure 1).

#### Histological analysis of the brain of *G. omarorum*

To reveal the structural organization of the brain regions under analysis, mainly those where newborn neurons migrate, some sections were counterstained with DAPI (Sigma) or ToPro3 (Molecular Probes, Grand Island, NY, USA) after immunohistochemistry. Also, serial sections of control animals (*N* = 2) were counterstained with fluorescent Nissl staining using Neuro Trace® (Molecular Probes) as described below (Supplementary Figure 1) and βIII-tubulin, HuC/HuD or tyrosine hydroxylase (TH; Supplementary Figure 2). For fluorescent Nissl counterstaining, free floating brain sections of a control animal were stained with Neuro Trace® at 1/150 dilution in PB for 20 min at room temperature and rinsed in PB with 0.1% Triton X-100 (10 min) followed by PB (3 × 5 min). For ToPro3 counterstaining, brain sections were immersed in ToPro3 for 10 min at a 1/4,000 dilution, followed by rinsing in PB (3 × 5 min). All sections were mounted in a sealing and anti-fading coverslipping solution containing polyvinyl alcohol (PVA, Sigma) and 1,4 diazabicyclo [2.2.2]octane (DABCO, Sigma) and prepared according Peterson lab protocol (http://neurorenew.com/wp-content/uploads/2014/12/pva2.pdf).

### Specific procedures

In order to evidence the migration and/or differentiation of newborn cells into the neuronal phenotype, we labeled derived cells using two protocols of thymidine analog administration. Then, we demonstrated their differentiation into the neuronal phenotype by demonstration of analog retention by CldU immunohistochemistry combined with immunohistochemical demonstration of neuronal markers' expression or labeling with neuronal tracers.

#### Thymidine analog labeling

Proliferating cells were labeled with 2.3 mg/ml 5-Chloro-2′-deoxyuridine thymidine (CldU, Sigma-Aldrich) dissolved in 0.7% sodium chloride, and administered i.p. at 20 μl/g of body weight, according to two procedures: (1) pulse administration: a single injection followed by post-thymidine analog survivals of 1 (*N* = 3), 7 (*N* = 2), or 30 days (*N* = 3); and (2) sequential administration: four daily injections followed by survivals of 90 (*N* = 2) or 180 days (*N* = 1).

#### *In vivo* neuronal tracer administration

To reveal the differentiation of cerebellar newborn cells into the neuronal phenotype, Neurobiotin tracer (Vector Laboratories, Inc., Burlingame, CA, USA) was applied *in vivo* at the molecular layer of the Cb, 86 or 176 days after repetitive CldU administration. Animals were anesthetized and the soft tissue over the rostralmost portion of the sagittal suture was carefully removed with the tip of a scalpel (Supplementary Figure 3A). Then, a small perpendicular and superficial incision was made in order to disrupt this portion of the sagittal suture and expose the dorsal surface of the rostral portion of CCb. After slightly cutting the cerebellar surface, crystals of Neurobiotin were applied with the tip of a needle (Supplementary Figure 3B). Finally, the wound was sealed with Histoacryl® (B. Braun Aesculap AG, Tuttligen, Germany), animals were allowed to recover from anesthesia and returned to their tanks.

#### Double immunohistochemistry (CldU label retention and neuronal markers' expression)

To break double-stranded DNA into single strands and expose the proliferation marker CldU to the antibodies, free floating tissue sections were first incubated in 2 N HCl (Baker, Phillipsburg, N.J. USA) in PB containing 0.3% Triton X-100 (Baker; PB-T) for 50 min at room temperature, followed by rinsing in PB (3 × 10 min). Then, sections were incubated for 1–2 days at 4°C in rat anti BrdU-CldU antibody (Accurate Chemical & Scientific Corporation, Westbury, NY, USA) at a dilution of 1:500 in PB-T, along with other primary antibody [rabbit anti doublecortin (Abcam, Cambridge, MA, USA) at 1/2,000 dilution, mouse anti HuC/HuD (Molecular Probes, Eugene, OR, USA) at 1:200 dilution, or rabbit anti TH (ThermoScientific, Waltham, MA USA) at 1/800 dilution]. Sections were rinsed in PB (3 × 10 min) and incubated in a mixture of donkey anti rat Cy5 secondary antibody (Jackson Immuno Research, West Grove, PA, USA) at 1:1,000 dilution and goat anti mouse Alexa 488 (Jackson Immuno Research) at 1:1,000 dilution in PB-T, for 90 min at room temperature. After rinsing in PB (3 × 10 min), sections were mounted in PVA/DABCO cover slipping solution. Negative controls involved the omission of CldU or of primary antibodies incubation; both controls resulted in no detectable staining (data not shown).

#### Simultaneous demonstration of CldU label retention and neuronal tracer labeling

In order to evidence the co-localization of CldU and Neurobiotin, sections were first pretreated in HCl and incubated in rat anti BrdU-CldU as described above. After rinsing in PB (3 × 10 min), sections were incubated during 90 min in donkey anti rat Cy5 at 1:1,000 dilution, together with streptavidin Cy3 (VECTOR) at a 1:500 dilution in PB-T. Sections were finally rinsed in PB (3 × 10 min) and mounted in PVA-DABCO coverslipping solution for immunofluorescence.

#### Image acquisition and processing

Most sections were imaged on a confocal system consisting of an Olympus BX61 microscope equipped with a FV300 confocal module and four lasers lines (405, 488, 543, and 633 nm) and the following objectives: 4x: UPlanFLN 0,13AN, 10x: UplanSAPO 0,40AN, 20x: UPlanFI 0,50AN, 40x: UPlanFI 0.75AN, and 60x: Plan ApoN 1,42AN objectives of the Confocal Microscopy Facility (IIBCE). Others were imaged on a Leica TCS SP5 II microscope equipped with four lasers lines (405, argon multiline 458, 476, 488, and 514 nm, and HeNe 543 and 633 nm), and HC PL FLUOTAR 5x/0.15, HC PL APO 20x/0.70 CS, HCX PL APO 40x/1.25-0.75 Oil, HCX PL APO 63x/1.40-0.60 Oil, HCX PL APO 63x/1.20 W CORR, and PL APO 100x/1.40-0.70 Oil CS objectives of the Confocal Microscopy Unit (Facultad de Medicina, UdelaR).

Acquisition settings were adjusted to ensure the completely dynamic range detection. Images of confocal planes were sequentially scanned with each laser line. Fifteen confocal planes, every 2 μm, were acquired in sections of levels L1–L3. For levels L4–L6, 15, 20, 25 to 30 confocal planes, every 0.5 or 1 μm were acquired. Post-processing of images was limited to small changes in the distribution histogram or background subtraction (Fiji Rolling ball radius: 50 pixels) in most cases.

Co-expression of markers was confirmed by orthogonal projections in the X-Z and Y-Z planes of the z-stacks at X-Y position of presumptive co-localization and/or visualization of the images corresponding to each channel of a single plane separately along with their overlay.

We used the same nomenclature and abbreviations as previously (Maler et al., 1991; Zupanc et al., 1996; Corrêa et al., 1998; Meek and Nieuwenhuys, 1998; Olivera-Pasilio et al., 2014).

#### Quantification

To quantify the amount of labeled cells per section in levels L1–L3, we constructed 3-D models of the sections from serial 2D low power confocal microphotographs with BioVis3D (R) as detailed in Iribarne and Castelló (2014). The boundaries of the olfactory bulb (OB), and internal cell layer were drawn and the location of every labeled nucleus was indicated in each plane of the 3D reconstruction with the tool “dot sets.” The spatial distribution of labeled nuclei was evidenced by making transparent the constructed models.

We also used BioVis3D to determine the location of labeled nuclei in the x and y axes. First, we established the crossing of the mid-line and dorsal border of the OB as the origin of the x and y axes (red lines in Supplementary Figure 1). Then, lines extending from each nucleus to the x and y axes (green and blue lines in Supplementary Figure 1) were drawn and their length was calculated by the software. The resulting values were normalized by the width and height of each region of interest at each studied level to calculate the relative distance of every nucleus.

For every level (L1–L3) of each chase duration (1, 7, and 30 days), the mean and standard deviation of the total amount of labeled nuclei per section were calculated. We also quantified the amount of labeled nuclei within and outside the internal cell layer of L1 and L2 (Supplementary Figures 4A,B), and within and outside the ventricular proliferation zone adjacent to the ventral and dorsal subdivisions of the ventral telencephalon of L3 (Supplementary Figure 4C).

Differentiation into the neuronal phenotype by demonstration of the expression of neuronal markers (HuC/HuD or TH) or retrograde Neurobiotin labeling of long term CldU label retaining newborn cells was quantified with the aid of Fiji-ImageJ Point tool. In z stacks of the brain regions of interest, CldU+ nuclei, and double labeled CldU+/HuC/HuD+, CldU+/TH+, or CldU/Neurobiotin cells were marked in selected equally spaced confocal planes to avoid over counting. The total amount of each class was registered and the percentages of CldU+ cells that expressed neuronal markers or were labeled with Neurobiotin were calculated.

#### Statistical analysis

We analyzed variations in the amount of CldU labeled cells per section using One-way ANOVA or Mann-Whitney *U*-test, after determining the variance of the samples with Kolmogorov-Smirnov test. Differences among groups were considered significant when *p* < 0.05.

## Results

### Spatial distribution of brain proliferation zones

We first analyzed the spatial distribution of brain proliferation zones in adult *G. omarorum* as a reference to depict the migration and/or differentiation of newborn cells. The spatial distribution of CldU labeled cells after a short survival (1 day) following the administration of a pulse of CldU (red dots in Figure 1) reproduced previous findings (Olivera-Pasilio et al., 2014). Briefly, most proliferating cells populated ventricular proliferation zones at the lining of the telencephalic (L2, L3, Figure 1), mesencephalic, diencephalic (L4, L5, Figure 1), and rhombencephalic (L6, Figure 1) ventricles. Outstanding: clusters of proliferating cells were found in all cerebellar divisions (L4, L5, Figure 1) and in the lateral-caudal pole of the ELL (L6, Figure 1). This spatial pattern allowed us to depict the boundaries of proliferation zones that were used as a reference to determine whether derived cells persist within the proliferation zones or migrate toward other regions or sub-regions at longer survival times (7–180 days).

### Migration of newborn cells

Some labeled cells remained within the proliferation zones after post CldU survival times between 7 and 180 days. Conversely, most of the CldU labeled cells were located at increasingly greater distances from the proliferation zones boundaries, according to the survival duration. This shift is exemplified in Figure 1 for survivals of 1 and 30 days (red and blue dots in Figure 1). The abundance of migrating newborn cells and the locations that they reached as a result of the migration varied among proliferation zones. In this work we focused on the brain regions where the migration process was more salient: the rostral telencephalon and OB, the TeO, the TS, and the Cb.

#### Olfactory bulb and rostral telencephalon

The sessile OB of *G. omarorum* shares the histoarchitecture of teleost's OB (Byrd and Brunjes, 1995; Kermen et al., 2013), consisting of a round core of small and densely packed cells, the internal cell layer (ICL) surrounded by three concentric diffuse layers: the external cell layer (ECL), the glomerular layer (GL), and the outer primary olfactory fiber layer (POFL) adjacent to the OB's surface (L1 and L2 in Supplementary Figure 1).

**Figure 2:**
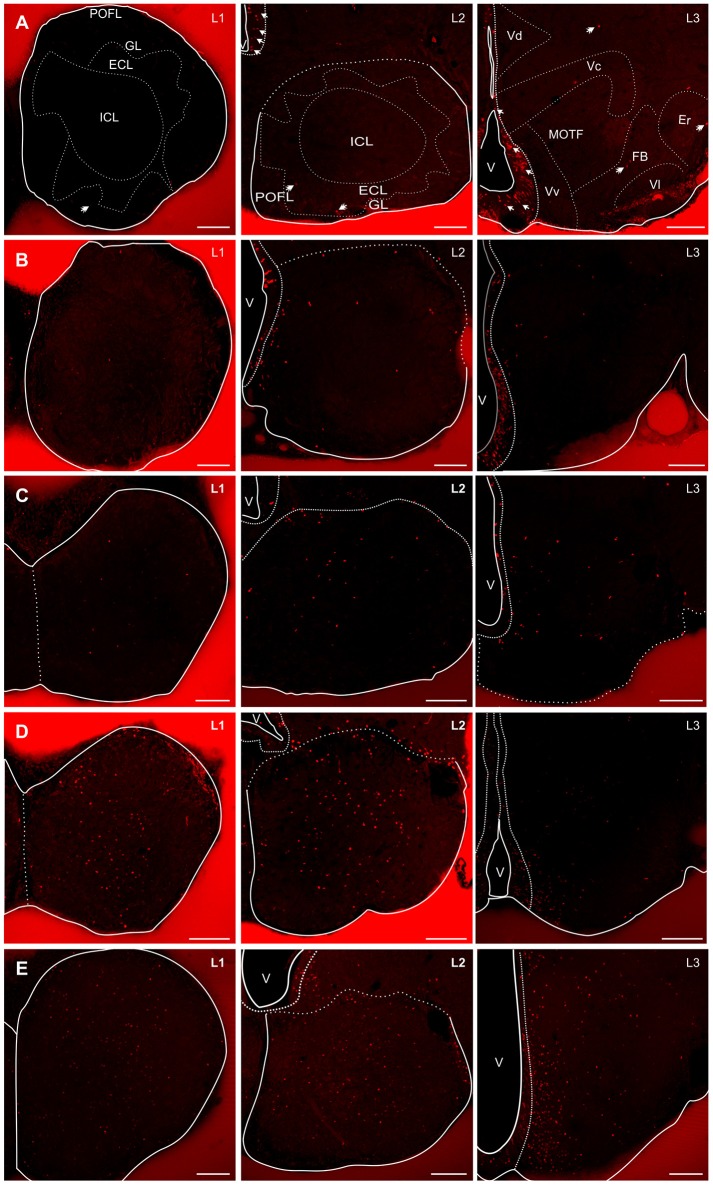
Spatial-temporal distribution of proliferating cells and derived cells in the rostral (L1) and caudal (L2) regions of the OB, and rostral subpallium (L3) in *G. omarorum*. Images correspond to microphotographs of frontal sections of the brains of animals treated with a single **(A–C)** or four daily doses **(D,E)** of CldU followed by 1 **(A)**, 7 **(B)**, 30 **(C)**, 90 **(D)**, and 180 **(E)** days of survival. The OB is almost devoid of CldU+ cells after short survivals (**A** L1, L2). Conversely, the lining of the rostral portion of the telencephalic ventricle, adjacent to the subpallium was populated by densely packed CldU labeled cells, conforming a clear proliferation zone (arrows in **A** L2, L3). From 1 to 30 days CldU+ cells appeared within the nerve tissue at progressively greater distances from the proliferation zones at L2 and L3. At longer chases (90 and 180 days), CldU+ cells also populate the rostral OB, and appeared to increase in number in L1–L3. ECL, External cell layer; Er, Rostral entopeduncular nucleus; FB, Forebrain bundle; GL, granular layer; ICL, Internal cell layer; MOTF, medial olfactory terminal field; POFL, V, Ventricle; Vc, Ventral telencephalon, central subdivision Vl, Ventral telencephalon, lateral subdivision. Scale bars: 100 μm.

**Figure 3:**
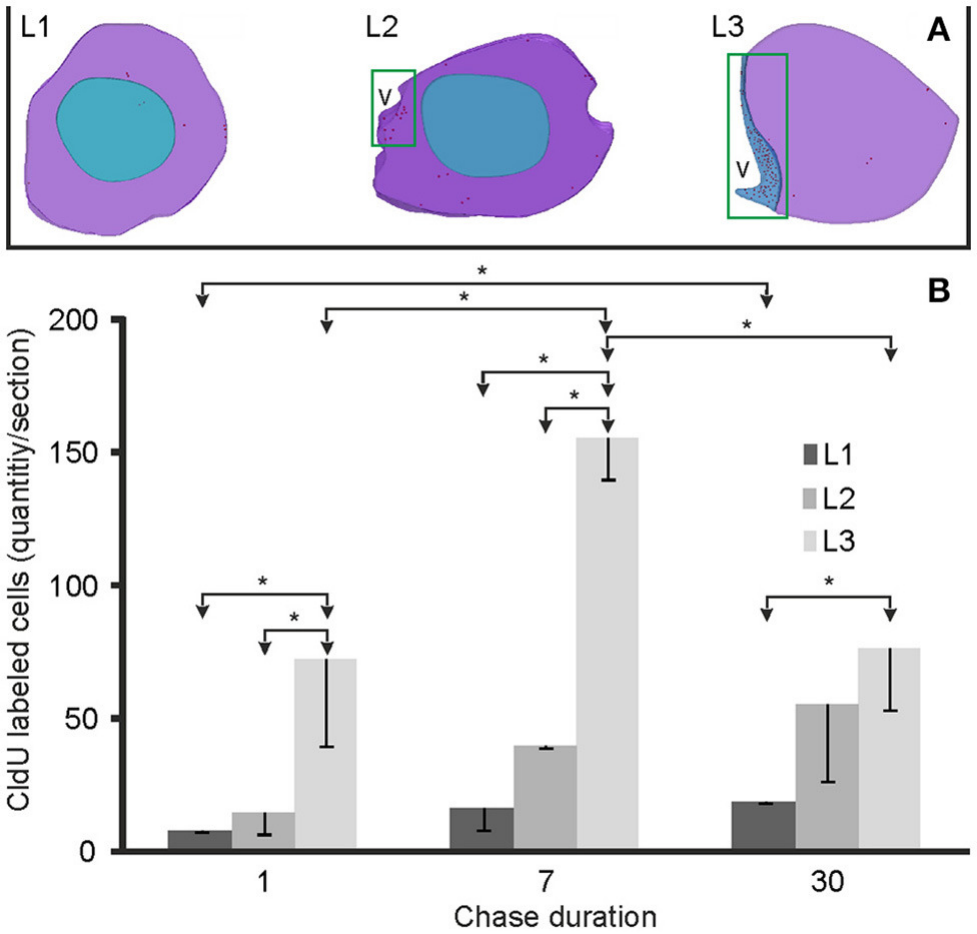
Spatial-temporal distribution and quantification of proliferating and derived cells within the rostral telencephalon. **(A)** The location of proliferating cells is represented by red dots in the 3D reconstructions of rostral (L1) and caudal (L2) olfactory bulb and rostral subpallium (L3). Proliferation zones were located at the ventricular lining indicated by green rectangles in L2 and L3. **(B)** Bar plots of the amount of labeled cells per section at the three levels studied (L1–L3) following 1 (*N* = 3), 7 (*N* = 2), and 30 (*N* = 3) days after the administration of a pulse of CldU. Values are expressed as the mean and standard deviation of the total amount of labeled cells per section. ^*^ <0.05, One-way ANOVA.

One day after a pulse of CldU, labeled cells were rare in the OB. Few and scattered proliferating cells were located at any of the layers of the rostral and medial part of the OB (red dots in L1 and L2, Figure 1: double arrows in AL1 and AL2, Figure 2; red dots in AL1 and AL2, Figure 3A). Conversely, densely packed CldU labeled cells were found at the lining of the most rostral portion of the telencephalic ventricle, nearby the caudal OB and the rostral region of the ventral (Vv) and dorsal (Vd) subdivisions of the subpallium. There, proliferating cells formed an extended ventricular proliferation zone (zone 1b, Olivera-Pasilio et al., 2014; red dots lining the ventricle at the zones indicated by the green rectangles in L2 and L3, Figures 1, 3A; arrows in AL2 and AL3, Figure 2). This rostral-caudal gradient in the amount of proliferating cells was confirmed quantitatively as the number of proliferating cells in the rostral and caudal part of the OB was significantly lower than in the rostral portion of the subpallium 1 day after CldU administration (Figure 3B).

Seven days after CldU administration, proliferating cells were also located predominantly at ventricular proliferation zones in the caudal portion of the OB and rostral subpallium (BL1–L3, Figure 2). Only few CldU labeled nuclei were placed throughout the OB layers or the subpallium. The number of labeled cells increased in all levels at this chase duration, but only significantly at L3. The rostral-caudal gradient was preserved but steeper (Figure 3B).

Thirty days after CldU administration, some proliferating cells remained within the boundaries of the proliferation zone 1b adjacent to the caudal OB (CL2, Figure 2) and rostral subpallium (CL3, Figure 2). Unlike shorter chases, some CldU labeled nuclei were located at considerable distances from the rostral region of the proliferation zone 1b, reaching the ICL of caudal and, in a less extent, rostral OB (CL2 and CL1, Figure 2). The amount of CldU labeled cells in L1 and L2 increased up to 2.4 and 3.7 times the values found 1 day after CldU administration, respectively. However, only L1 showed significant differences in the amount of CldU labeled cells between both chases. Conversely, the amount of CldU labeled cells decreased significantly in L3 from 7 to 30 days (Figure 3B). Few CldU labeled nuclei were found lateral to the caudal zone of 1b (adjacent to the subpallium), reaching Vd, Vv, MOTF, and medial zone of FB (CL3, Figure 2).

CldU label retaining cells spilled out the boundaries of the ICL of the caudal OB at a 90 day chase (Figure 2, EL2) whereas most labeled cells remain within the ICL boundaries of the ICL of the rostral OB (Figure 2, EL1). At 180 day chase the spilled out of labeled cells from the ICL of the rostral OB to the surrounding layer was evident (Figure 2, FL1).

The amount of CldU label retaining cells in the lateral zone of the subpallium increased considerably 90 and 180 day after CldU administration. At 90 days newborn cells predominate in the ventral zone, whereas at 180 days were distributed along all the dorso-ventral extension of the subpallium.

#### Cerebellum

All the divisions of the Cb of *G. omarorum* (CCb, valvula cerebelli -VCb, and caudal lobe, including the eminentia granularis anterior -EGa- and posterior -EGp-), are organized in three layers, molecular, ganglionic, and granular, as exemplified in Supplementary Figure 5A. The ganglionic layer consists of Purkinje and eurydendroid cells. Eurydendroid cells, which are the origin of Cb efferents, are not aggregated in nuclei unlike other vertebrates (Finger, 1978).

Remarkable migrations processes both for the abundance of migrating cells as well as for their final destination, were detected in all cerebellar divisions as previously described (Olivera-Pasilio et al., 2014). Proliferation zones were circumscribed to a single cerebellar layer or even a region within a single cerebellar layer (red dots in L4 and L5, Figure 1).

The proliferation zone of the CCb, evidenced by the distribution of proliferating cells at 1 day chase, occupied the medial zone of its molecular layer (CCb-mol; red dots in L4, Figure 1). Thirty days after CldU administration, derived cells were mainly found at the granular layer of CCb (CCb-gra; blue dots in L4, Figure 1). However, scarce and intensely labeled CldU cells remained within the proliferation zone's boundaries.

In the VCb a conspicuous proliferation zone occupied all the extension of the molecular layer (VCb-mol). Proliferating cells were more densely packed near the dorsal-medial region of VCb-mol and migrating cells were sparsely and homogeneously found in all the extension of the granular layer (VCb-gra).

The proliferation zone of the caudal lobe of the Cb occupied the granular layer of the medial granular eminence (EGm-gra) while derived cells were located at the granular layer of the posterior granular eminence (EGp-gra) as shown in Olivera-Pasilio et al. (2014).

#### Tectum opticum

The TeO is a multimodal integration center which is interconnected with the torus longitudinalis forming a Cb-like structure (Bell, 2002). In *G. omarorum*, it is a hollow hemispheric shaped structure. It has a cortical histoarchitecture and consists of seven cellular and fibrillary alternating layers (Supplementary Figure 5B; Meek and Nieuwenhuys, 1998). Proliferating cells were located at the dorsal-medial and ventral-lateral border of the caudal pole of TeO, forming a horseshoe shaped ribbon-like proliferation zone. One day after CldU administration, proliferating cells were distributed in all TeO layers (red dots in L4 and L5, Figure 1), though appeared to be more densely packed in deeper layers as the stratum periventriculare (SPV). At longer chases, CldU label cells occupied more superficial layers of TeO, also extending beyond the proliferation zones' boundaries in the rostral direction (blue dots in L4 and L5, Figure 1).

One hundred eighty days after CldU administration, derived cells were located ~100 μm away from the dorsal region of the proliferation zone at rostral levels of the caudal TeO. These long labeled retaining cells were grouped mainly at the SPV and SAC layers, and less frequently reached the SGC (L5, Figure 1).

#### Torus semicircularis

The TS of *G. omarorum* is a spherical protrusion of the mesencephalic tegmentum beneath the TeO. It is a layered structure which receives octavolateral inputs from rhombencephalic primary brain centers (Meek and Nieuwenhuys, 1998). One day after CldU administration proliferating cells were located at the medial and lateral borders of the caudal pole of TS (red dots in L5, Figure 1), forming a ring-shaped proliferation zone. At longer chases derived cells appeared diffusely distributed at the caudal pole of TS (blue dots in L5, Figure 1).

#### Electrosensory lateral line lobe

The ELL is the primary relay nucleus of electrosensory pathways. It is a layered cerebellum-like structure (Supplementary Figure 5C; Meek and Nieuwenhuys, 1998) in which principal cells project to contralateral rhombencephalic and mesencephalic structures (praeminentialis nucleus and TS) which project back to the ELL, either directly or indirectly through Cb. At 1 day chase duration, proliferating cells were mainly located at the lateral-caudal border of cellular layers of ELL, and showed a medial-lateral and rostral-caudal gradient (red dots in L6, Figure 1). At longer chases, ELL CldU label retaining cells were extensively and diffusely distributed (blue dots in L6, Figure 1).

### Neuronal differentiation and insertion into neuronal circuits

With the aim of studying neuronal differentiation and neurogenic capacity of *G. omarorum* adult brain proliferation zones, we analyzed the expression of neuronal markers, including the early neuronal markers DCX, and HuC/HuD or markers of further differentiation into the neuronal phenotype as tyrosine hydroxylase (TH), in CldU label retaining cells after post thymidine analog administration chases ranging from 7 to 180 days. We also assessed the insertion of newborn neurons into pre-existing neural circuits of the CCb by “*in vivo*” retrograde labeling of granule cell after Neurobiotin administration at the molecular layer of CCb.

#### Neuronal differentiation in the olfactory bulb and rostral telencephalon evidenced by co-localization of CldU and HuC/HuD, DCX, or TH

One day after thymidine analog administration, DCX labeled a net of processes beneath the ventricular proliferation zone adjacent to the medial and dorsal-medial zone of the telencephalic proliferation zone 1b (inset, Supplementary Figure 6A). We only found CldU-DCX double labeled cells adjacent to the ventral region of this proliferation zone (Supplementary Figure 6B) as evidenced by the overlay of CldU and DCX signals corresponding to a single confocal plane (Supplementary Figure 6C) or by orthogonal projections of a z stack (Supplementary Figure 6D). At this chase duration, we did not find CldU-DCX or CldU-HuC/HuD double labeled cells in any other brain region.

**Figure 4:**
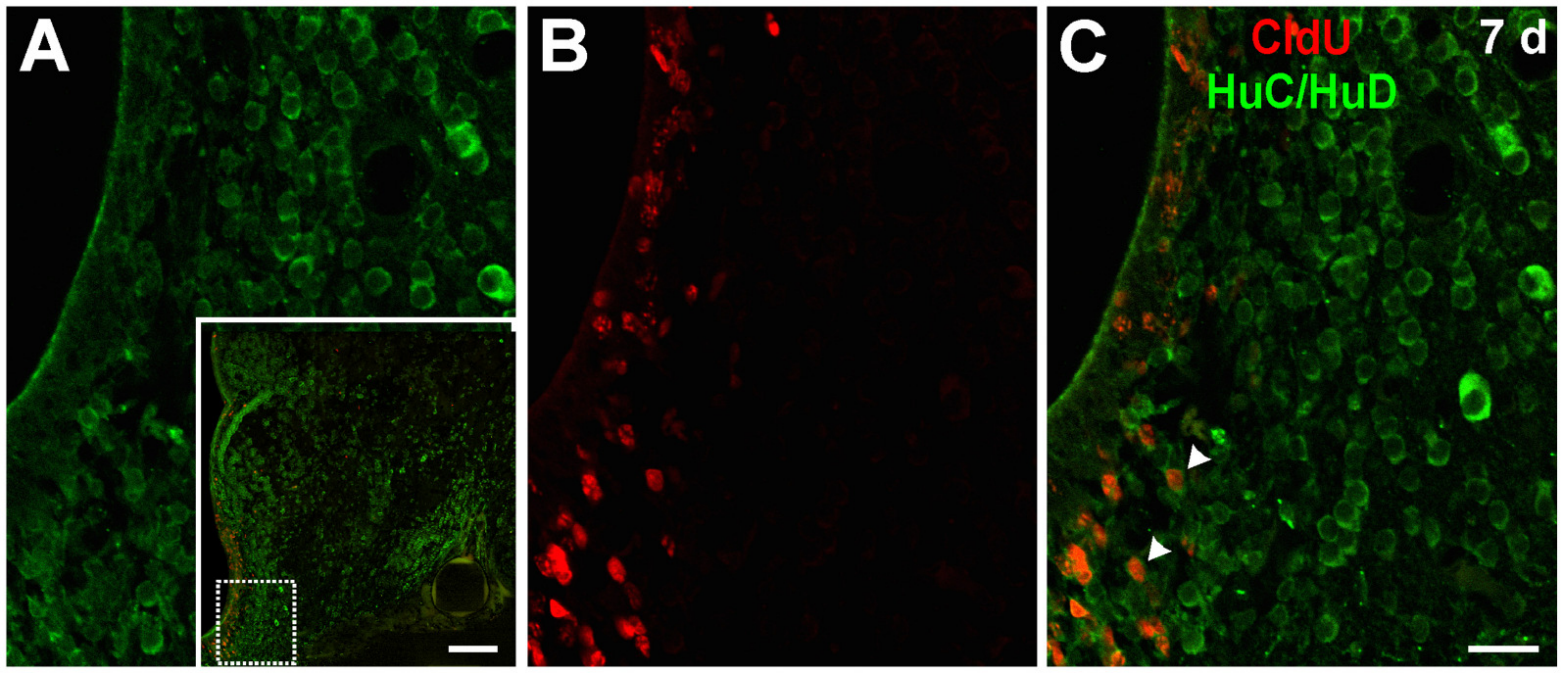
Neuronal differentiation in the subpallium at 7 days after the administration of a pulse of CldU demonstrated by co-localization with HuC/HuD. **(A)** Confocal microphotographs of the ventricular lining at the region of the rostral subpallium indicated by the rectangle in the panoramic view (inset). HuC/HuD and CldU labeling are shown in green and red channels in the microphotographs of a single plane shown in **(A,B)**, respectively. The overlay of both channels is shown in **(C)**. Arrowheads indicate CldU labeled retaining cells also expressing the neuronal marker HuC/HuD. Scale bars: (inset) 100 μm; **(A–C)** 20 μm.

Seven days after CldU administration, some cells at the ventral part of the subventricular zone of the rostral subpallium were CldU-HuC/HuD double labeled (Figure 4), but not at the rostral or caudal zones of the OB. Unexpectedly, we did not find any CldU-DCX double labeled cell at this chase duration.

**Figure 5:**
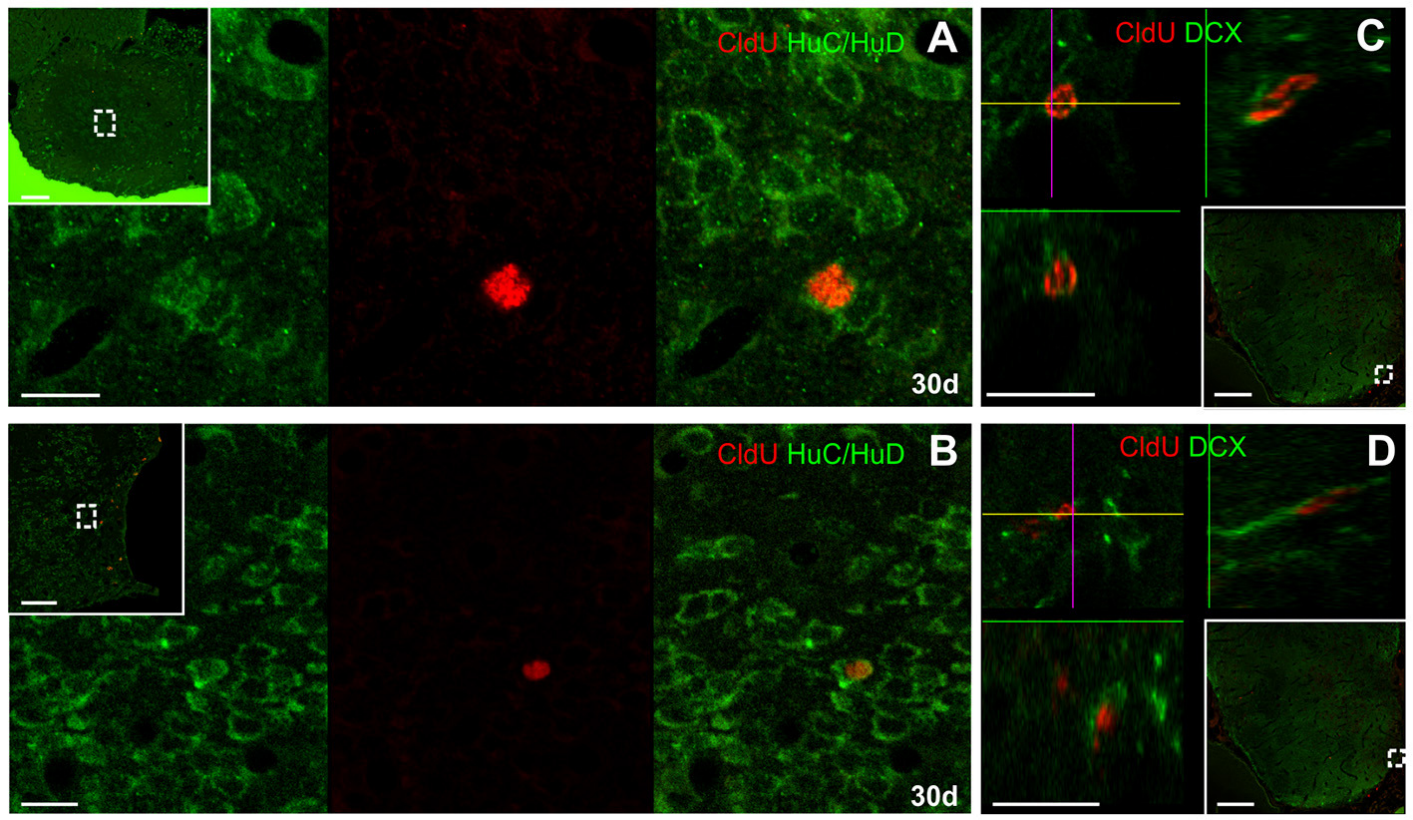
Neuronal differentiation in the olfactory bulb and rostral subpallium at 30 days after the administration of a pulse of CldU demonstrated by co-localization with HuC/HuD and DXC. **(A,B)** Confocal microphotographs of the caudal OB and rostral subpallium at the regions containing double labeled cells indicated by the dotted squares in the panoramic views (insets), respectively. HuC/HuD and CldU labeling are shown in green (left) and red (middle) channels; the right image corresponds to the overlays of both channels. **(C,D)** *x-z* and *y-z* orthogonal projection of stacks of 35 confocal planes scanned every 0.5 μm at the lines crossing at the cell of interest, confirming the co-localization of CldU and DCX in the rostral subpallium at the positions indicated by the dotted rectangles in the panoramic views (insets). Scale bars: **(A,B)** 10 μm; **(C,D)** 5 μm; insets, 100 μm.

After a 30 day chase, CldU label retaining cells expressed HuC/HuD at the ICL of the caudal OB (Figure 5A) and the ventral region of the subventricular zone of the rostral subpallium (Figure 5B). CldU-HuC/HuD double labeled cells' nuclei were round and the chromatin either densely (Figure 5A) or loosely (Figure 5B) packed. Some newborn CldU labeled cells with fusiform shape and elongated nuclei with densely packed chromatin also express DCX (Figures 5C,D).

**Figure 6:**
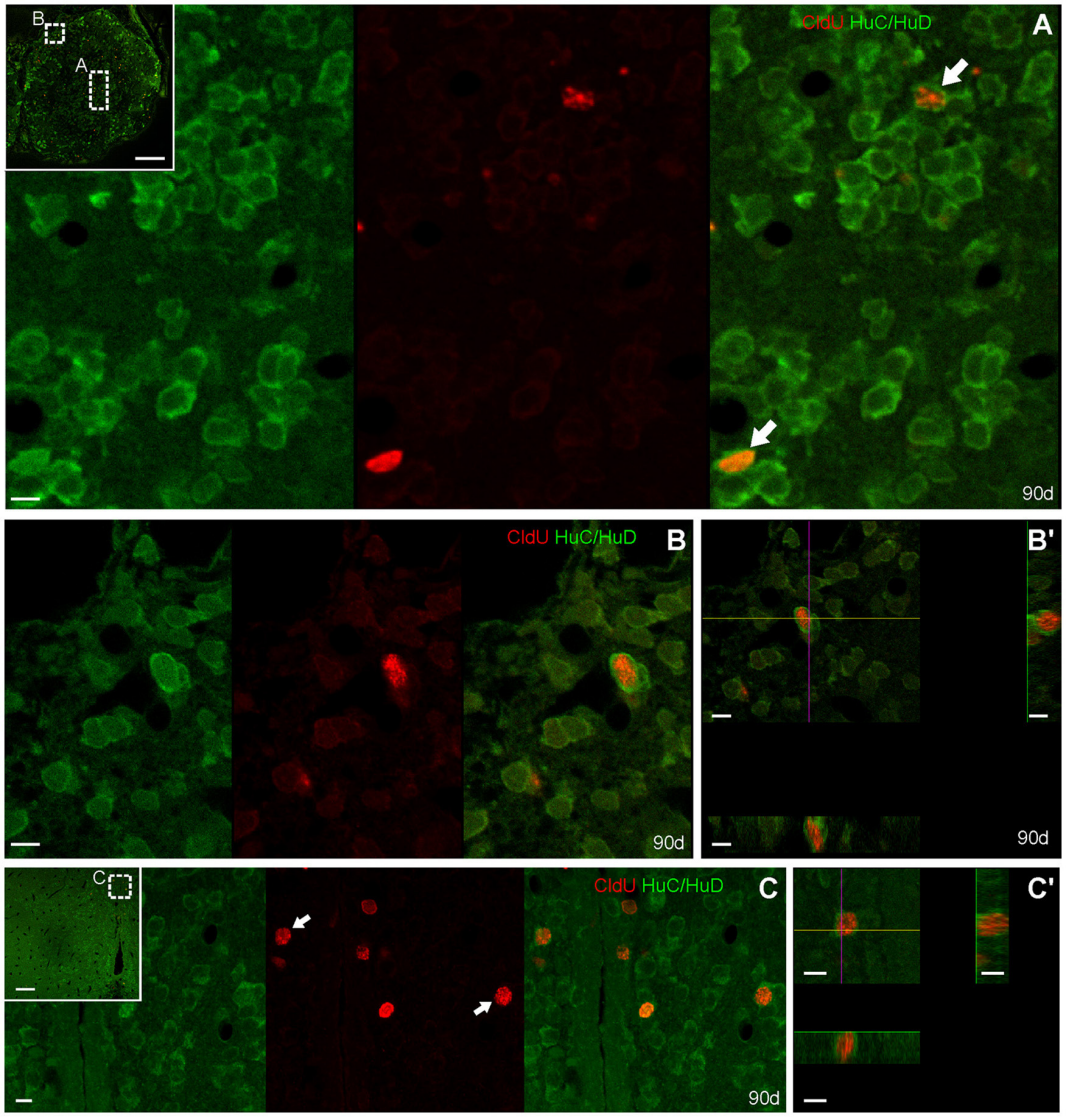
Neuronal differentiation in the olfactory bulb and rostral subpallium at 90 days after four daily injections of CldU demonstrated by co-localization with HuC/HuD. Confocal microphotographs of the olfactory bulb **(A,B,B**′**)** and the rostral subpallium **(C,C**′**)** at the level indicated by the dotted rectangles in the panoramic view (insets) to evidence double labeled cells. HuC/HuD and CldU labeling are shown in green and red channels of single scans, respectively. Co-localization is confirmed by the overlay of both channels, or by the *x*-*z* and *y*-*z* orthogonal projections of stacks obtained from 19 planes, every 0.5 μm **(B**′**)**, or 15 planes, every 0.5 μm **(C**′**)**.

Double labeled CldU-HuC/HuD cells were found at the ICL (Figure 6A) and the dorso-medial zone (Figure 6B) of the rostral OB 90 days after CldU administration. All CldU-HuC/HuD double-labeled cells had ovoid to round nuclei surrounded by a thin cytoplasm without evident cellular processes. At 90 days CldU-HuC/HuD double-labeled were also found at L3 (Figure 6C).

At 90 days, double labeled CldU-DCX cells were only found at the dorsal-medial zone of the rostral level of OB (data not shown).

**Figure 7:**
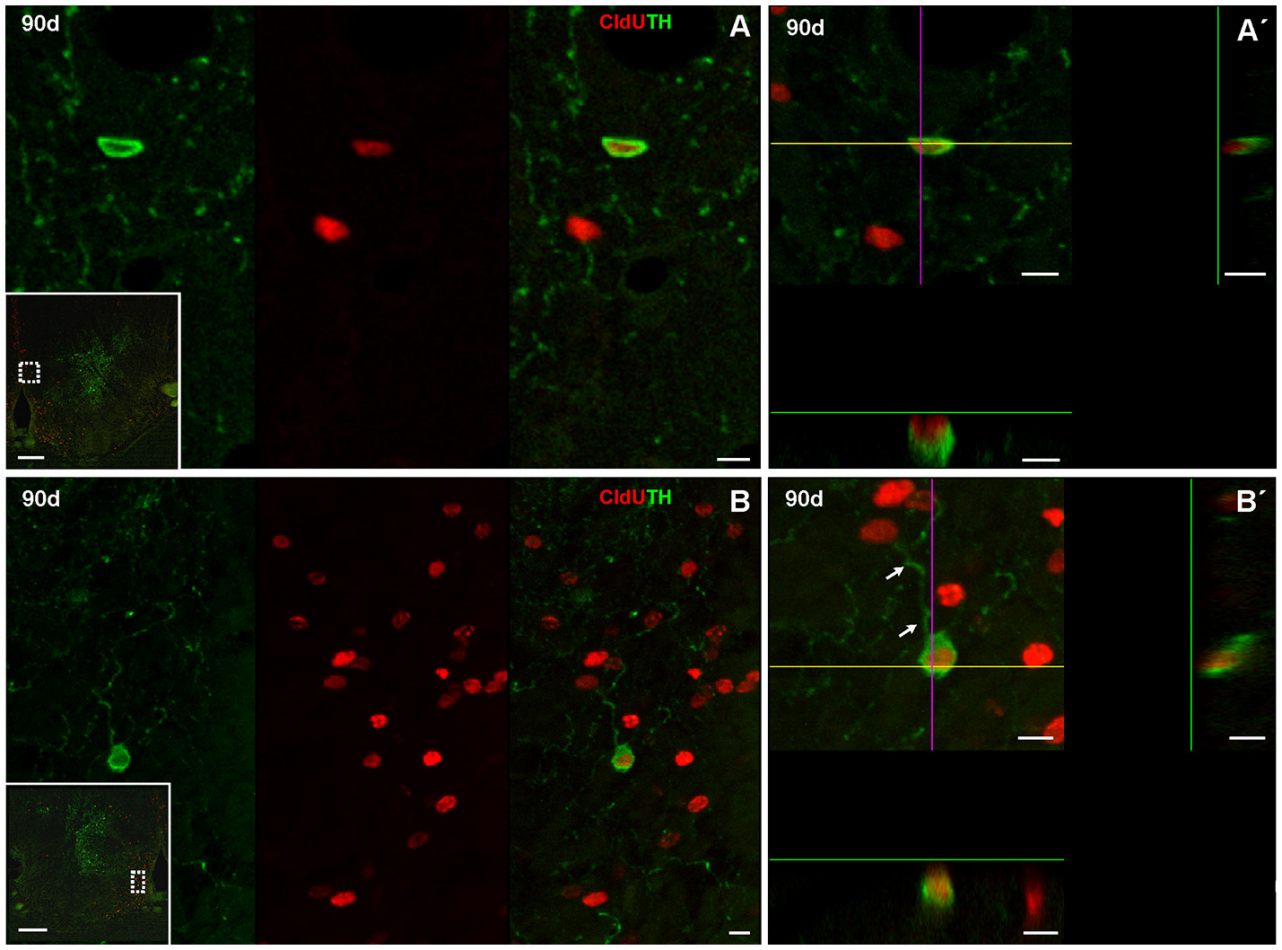
Neuronal differentiation in the rostral subpallium at 90 days after after four daily injections of CldU demonstrated by co-localization with TH. **(A,B)** Confocal microphotographs of the subpallium at the regions indicated by the dotted rectangles in the panoramic views (insets). Co-localization is demonstrated by TH and CldU labeling of a single confocal plane and the overlay of both images **(A,B)**. Co-localization is also confirmed by the *x-z* and *y-z* orthogonal projections of 16 confocal planes, every 0.5 μm **(A**′**)** or 45 confocal planes, every 0.5 μm **(B**′**)**. Arrows indicate neuronal processes of a newborn neuron. Scale bars: **(A,B)** 20 μm; **(A**′**,B**′**)** 5 μm; insets 100 μm.

At this chase duration CldU labeled cells also express TH at the ventricular or sub ventricular zone of the rostral subpallium (Figure 7). Some of these cells showed a thin cytoplasmic halo without cellular processes (Figures 7A,A′) whereas others had more expanded cytoplasm from which thin and short cellular processes with few branching points emerge (Figure 7B, arrowheads in Figure 7B′).

**Figure 8:**
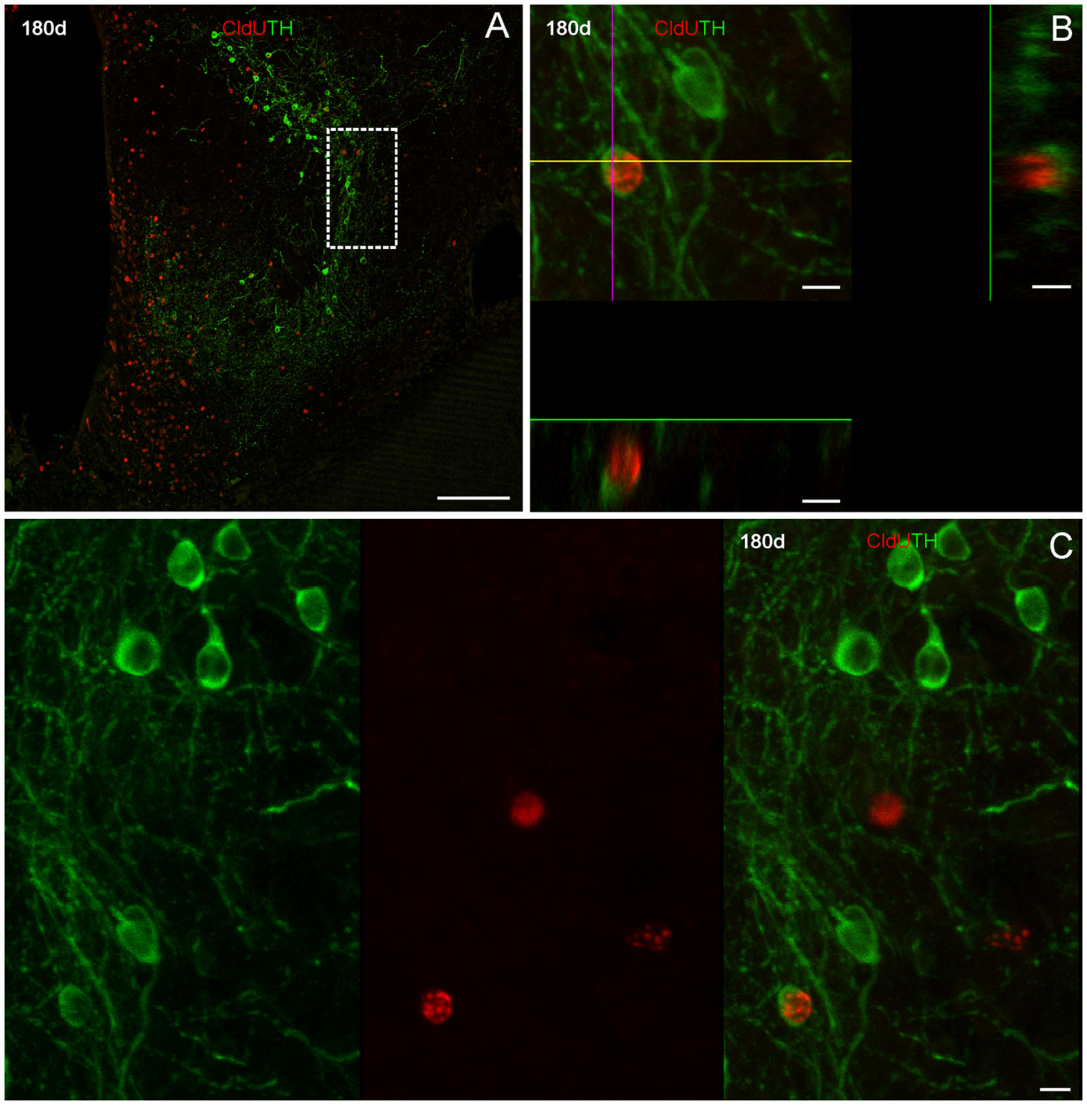
Neuronal differentiation in the rostral subpallium at 180 days after four daily injections of CldU demonstrated by co-localization with TH. **(A)** Maximal intensity projection of 15 confocal planes taken every 2 μm at the rostral subpallium. **(B)** *x-z* and *y-z* orthogonal projection of 25 confocal planes, every 2 μm, confirming the co-localization of CldU and TH in a cell at the region indicated by the dotted rectangle in **(A)**. **(C)** Co-localization of TH and CldU is also confirmed by the overlay of the sequentially acquired channels a single plane. Scale bars **(A)** 100 μm; **(B,C)** 5 μm.

**Figure 9:**
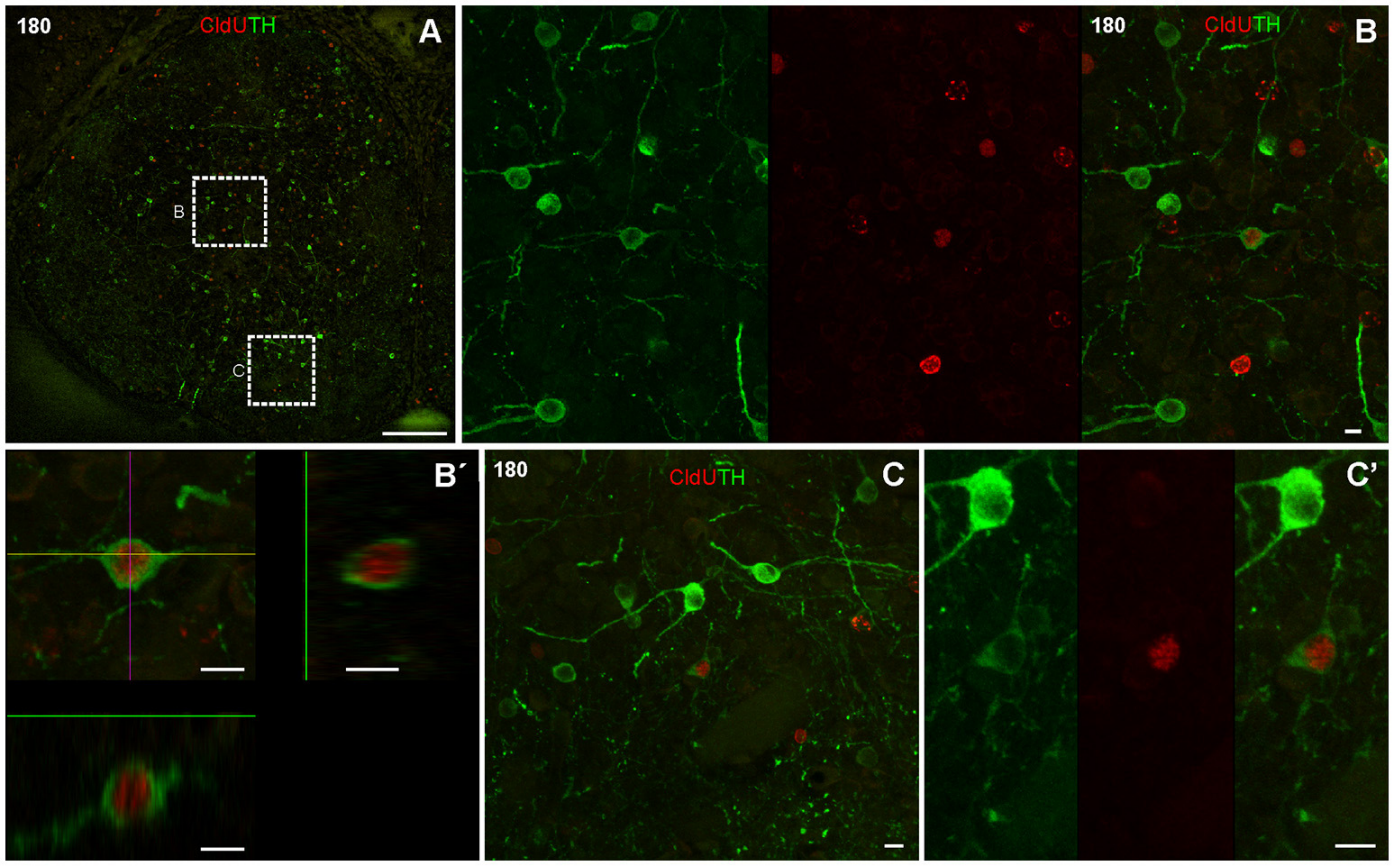
Neuronal differentiation in the caudal olfactory bulb at 180 days after four daily injections of CldU demonstrated by colocalization with TH. **(A)** Topographic confocal image of a frontal section of the caudal olfactory bulb. The rectangles indicate the locations at which higher power confocal images were acquired **(B,B**′**,C,C**′**)**. Images corresponding to both channels of a single confocal plane and the overlay at the regions indicated by the dotted rectangles in **(A)** are shown in **(B,C**′**)**. **(B')** x-z and y-z orthogonal projection of 22 confocal planes, every 1 μm, confirming the colocalization of CldU and TH in the same cell as in **(B)**. Scale bars: **(A)** 50 μm; **(B–C')** 5 μm.

**Figure 10:**
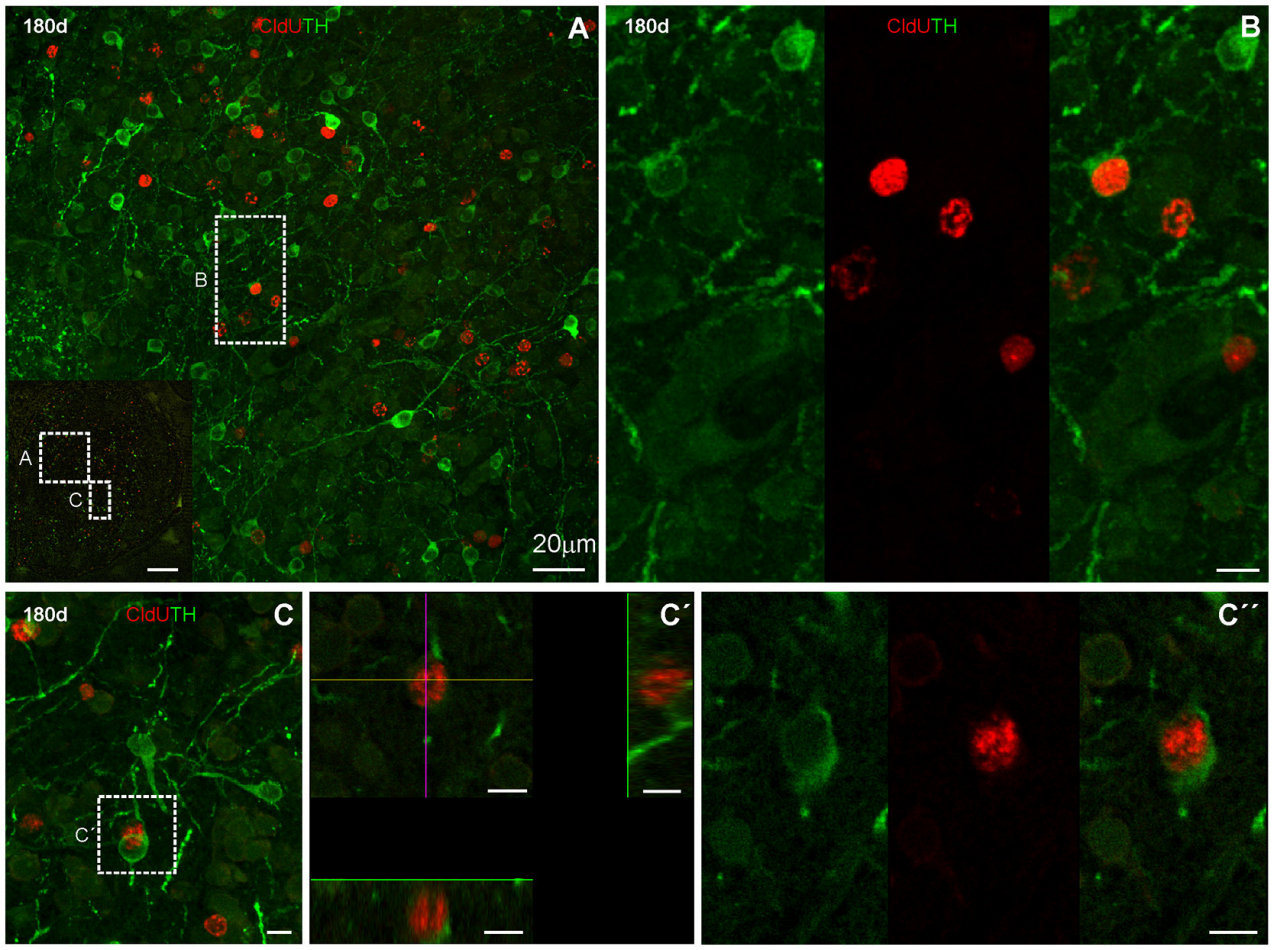
Neuronal differentiation in the rostral olfactory bulb at 180 days after four daily injections of CldU demonstrated by co-localization with TH. Microphotograph at the region of the rostral OB indicated by the rectangle in the inset in **(A)**. **(B)** Higher power images of both channels and the overlay of the region containing a cell of interest at the region indicated by the dotted square in the inset in **(A)**. **(C)** Maximal intensity projection of a stack of 17 confocal planes, every 0.5 μm. The rectangle indicates the location of a cell which co-localization was demonstrated by *x-z* and *y-z* orthogonal projections of the same stack **(C**′**)** as well as each channel and overlay of the sequentially acquired channels a single plane **(C**′′**)**. Scale bars: **(A)** 20 μm; (**B**, Inset in **A**) 100 μm; **(B,C**′′**)** 5 μm.

One hundred eighty days after CldU administration, double labeled CldU-TH cells were found in the rostral subpallium, far from the proliferation zone (Figure 8), and at both, caudal (Figure 9) and rostral (Figure 10) levels of the OB. Some newborn cells had an ovoid nucleus surrounded by a thin cytoplasmic halo without cellular processes, as those found lateral to the subpallial proliferation zone (Figures 8B,C). Many double labeled cells showed a larger cytoplasmic halo from which two or three relatively thick cellular processes emerged from opposite regions of the cell body. These cells resembling bipolar or multipolar neurons were located at the ICL of caudal (Figures 9B,B′,C,C′, Supplementary Video 1) and rostral (Figures 10C,C′,C″) OB. In other cells at the ICL of the rostral OB, a relatively large nucleus occupied an asymmetric location respect to a very thin cytoplasmic halo, resembling migrating neuroblasts (Figures 10A,B). All double labeled CldU-TH cells were intermingled with cells with single labeled CldU+ nuclei or TH+ cytoplasm.

According to the quantitative analysis, almost 0.5% of long term CldU retaining new born cells at the lateral regions of the subpallium (L3) expressed the mature neuronal marker TH+ after a 180 day chase. The fraction of CldU labeled cells that also expressed TH reached almost 1% and more than 1.5% of the total amount of CldU labeled cells at the caudal (L2) rostral (L1; Table 1).

**Table 1:** Quantitative analysis of neuronal differentiation in the brain of adult *G. omarorum*.

	**Brain region**	**90 days**				**180 days**			
		**CldU+**	**Double**	**%**	***N***	**CldU+**	**Double**	**%**	***N***
TH	OB (L1)	n.d.	n.d.	n.d.		434	7	1.61	1
	OB (L2)	n.d.	n.d.	n.d.		317	3	0.94	1
	Subpallim (L3)	419	2	0.47	1	n.d.	n.d.	n.d.	1
HuC/HuD	OB (L1)	51	26	50.98	1	n.d.	n.d.	n.d.	
	TeO (L4)	n.d.	n.d.	n.d.		519	403	77.65	2
	TeO (L5)	88	43	48.86	1	n.d.	n.d.	n.d.	
	TS (L4)	n.d.	n.d.	n.d.		142	124	87.32	1
	TS(L5)	18	54	33.33	1	354	253	71.46	2
	CCb (L4)	140	80	57.14	1	n.d.	n.d.	n.d.	
Nb	CCb (L4)	727	6	0.82	1	373	34	9.11	1

*Newborn cells retaining CldU after 90 or 180 days chases and co-expressing CldU and tyrosine hydroxylase (TH) or HuC/HuD, and newborn cells retaining CldU after 90 or 180 days chases and retrogradely labeled with Neurobiotin (Nb) were quantified in 1–4 z stacks of sections corresponding to the studied levels (intermediate olfactory bulb, OB; L1, caudal OB, L5 subpallium; L3, intermediate and, caudal tectum opticum (TeO, L4, and L5); intermediate and caudal torus semicircularis (TS, L4, and L5), and rostral corpus cerebelli (CCb, L5) of four specimens of G. omarorum. Results are expressed as the total amount of CldU labeled cells (CldU+), double labeled cells (Double), and as the fraction of long term CldU label retaining cells that also express one of the neuronal markers or were retrogradely labeled with Neurobiotin (%)*.

#### Neuronal differentiation in the corpus cerebelli and cerebellum-like structures evidenced by co-localization of CldU and HuC/HuD

After a short thymidine analog survival (7 days), we did not find HuC/HuD expression in CldU labeled cells. However, longer survivals (30, 90, and 180 days) allowed us to evidence the differentiation of derived cells into neurons in the CCb and other Cb-like structures.

##### Corpus cerebelli

We did not find CldU-DCX double labeled cells in CCb-mol 1 or 7 days after CldU administration, even though DCX processes were abundant at the CCb-mol (running parallel to the cerebellar surface in the medial-lateral direction) and the Purkinje cells' layer (running parallel to the cerebellar surface in the rostral-caudal direction; Supplementary Figure 7).

**Figure 11:**
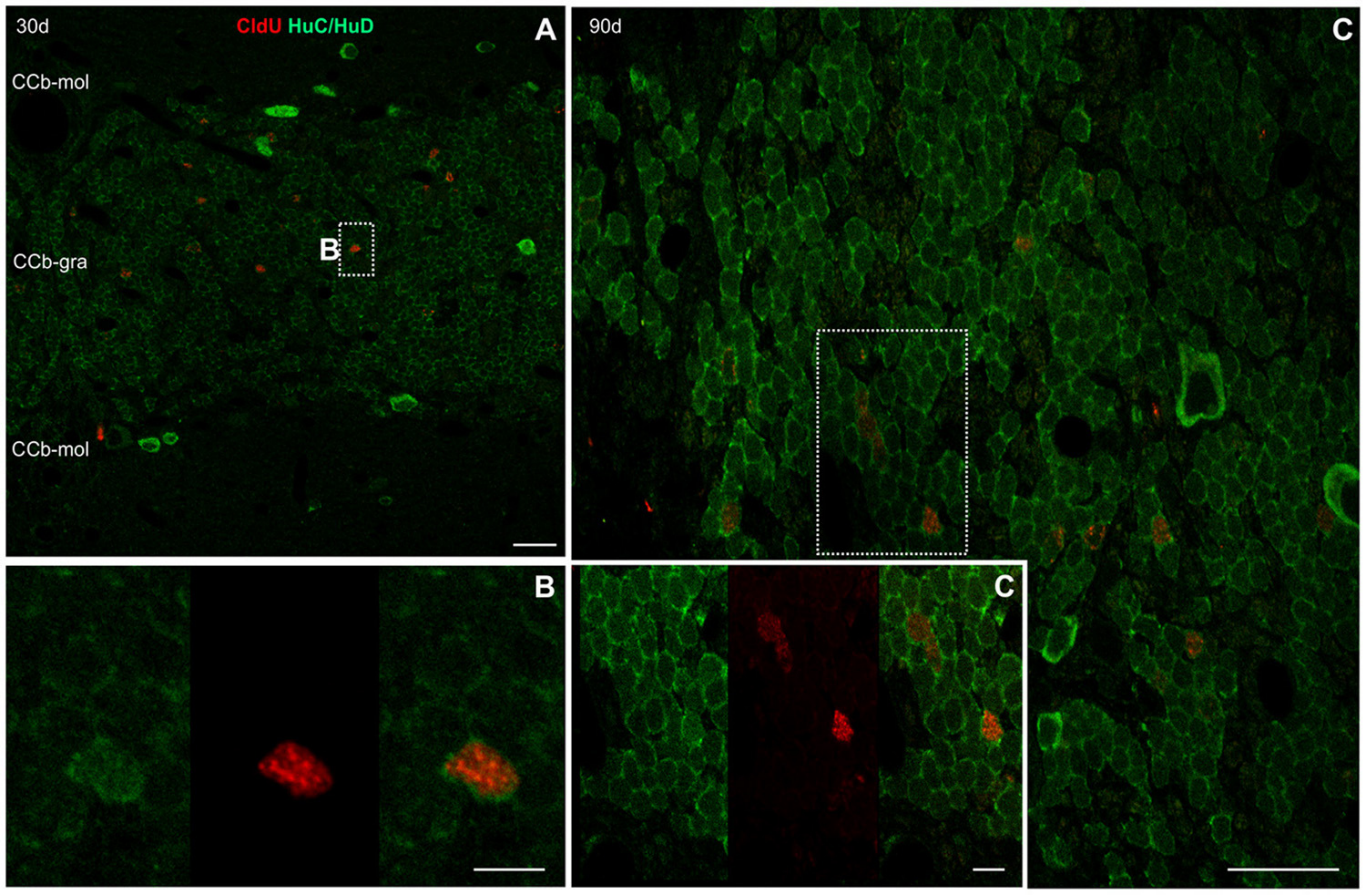
Neuronal differentiation in the corpus cerebelli at 30, 90, and, 180 days after four daily injections of CldU demonstrated by co-localization with HuC/HuD. **(A)** Low power microphotograph at the region of the corpus cerebelli. The dotted rectangle indicates location of a double labeled cell, as illustrated by the microphotographs of each channel and the overlay **(B)** in the inset in **(A)**. **(C)** Higher power image of a stack of 25 confocal planes, every 0.5 μm. The rectangle indicates the location of a cell which co-localization was demonstrated by the *x-z* and *y-z* orthogonal projections of the same stack **(C')** as well as each channel and overlay of a single plane **(C**″**)**. Scale bars: **(A)** 20 μm; (**B**, Inset in **A**) 100 μm; **(B,C”)** 5 μm.

Thirty days after CldU administration, scarce CldU labeled cells at the CCb-gra also expressed HuC/HuD (Figure 11A). These double labeled cells were small, with an ovoid or polygonal nucleus and granular CldU labeling of varied intensity, surrounded by a thin polygonal cytoplasmic halo (Figure 11B). Double labeled cells were intermingled with a myriad of HuC/HuD+ cells of the CCb-gra.

Double-labeled CldU-HuC/HuD cells with similar labeling characteristics were found at the CCb-gra 90 (Figures 11C,C′) and 180 days (data not shown) after CldU administration.

Purkinje and/or eurydendroid cells show a marked expression of HuC/HuD but did not showed co-localization with CldU at any of the chases studied.

##### Tectum opticum and torus semicircularis

Similar to the CCb, we did not find double labeled CldU-DCX newborn cells in the TeO at any of the chases studied, even though bundles of DCX processes were abundant beneath the dorsal-medial tectal proliferation zone (Supplementary Figure 8).

**Figure 12:**
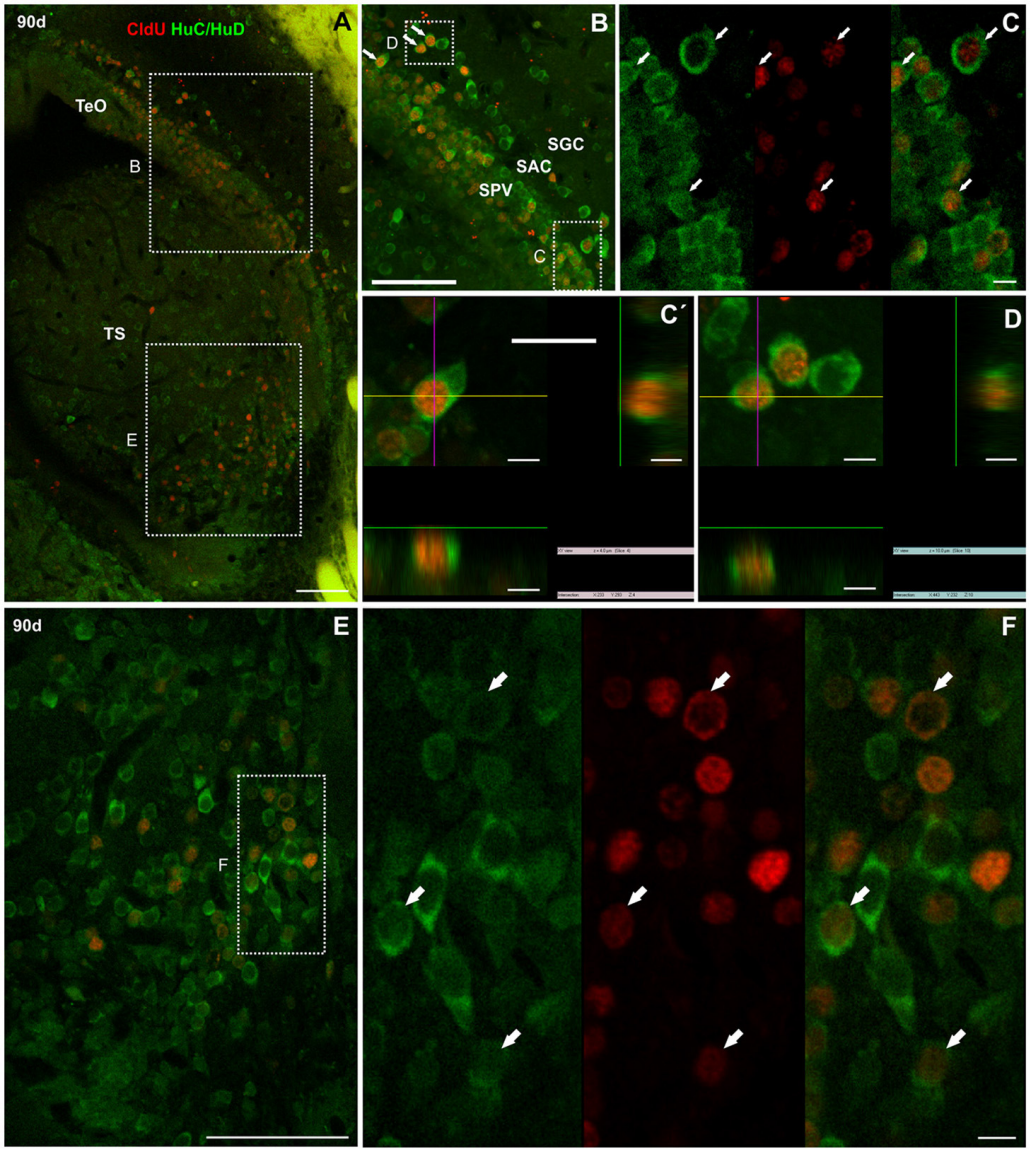
Neuronal differentiation in the tectum opticum and torus semicircularis at 90 and 180 days after four daily injections of CldU demonstrated by co-localization with HuC/HuD. **(A)** Maximal intensity projections of confocal microphotographs of a *z* stack (20 μm, every 0.5 μm) at the caudal pole of the tectum opticum (TeO) and torus semicircularis (TS). The dotted rectangles indicate location of two regions of interest observed at higher magnification in **(B,E)**. Double labeling of tectal cells **(B,C,C**′**,D)** was confirmed by the microphotograph of each channel and of the overlay **(C)** as well as the *x*-*z* and *y*-*z* projection of two stacks **(C**′**,D)**. Double labeling of toral cells is demonstrated in **(F)** by microphotographs of each channel and of the overlay of the region indicated by the dotted rectangle in **(E)**. Scale bars: **(A,B,E)** 50 μm; **(C,C**′**,D,F)** 5 μm.

Ninety days after CldU administration, long term label retaining cells also expressed HuC/HuD at the deeper layers of the caudal pole of the TeO (Figure 12). Double labeled cells were found in all dorsal-ventral extension of the stratum periventriculare (SPV), and in lesser density at the boundaries between stratum album centrale (SAC) and stratum griseum centrale (SGC). Most double labeled cells had round to ovoid nucleus surrounded by a thick cytoplasmic halo without cellular processes (Figures 12A–D). Likewise, CldU label retaining cells also expressing HuC/HuD with round morphology were found at the ventral region of the TS. Double labeled cells showed a round morphology (Figures 12E,F).

**Figure 13:**
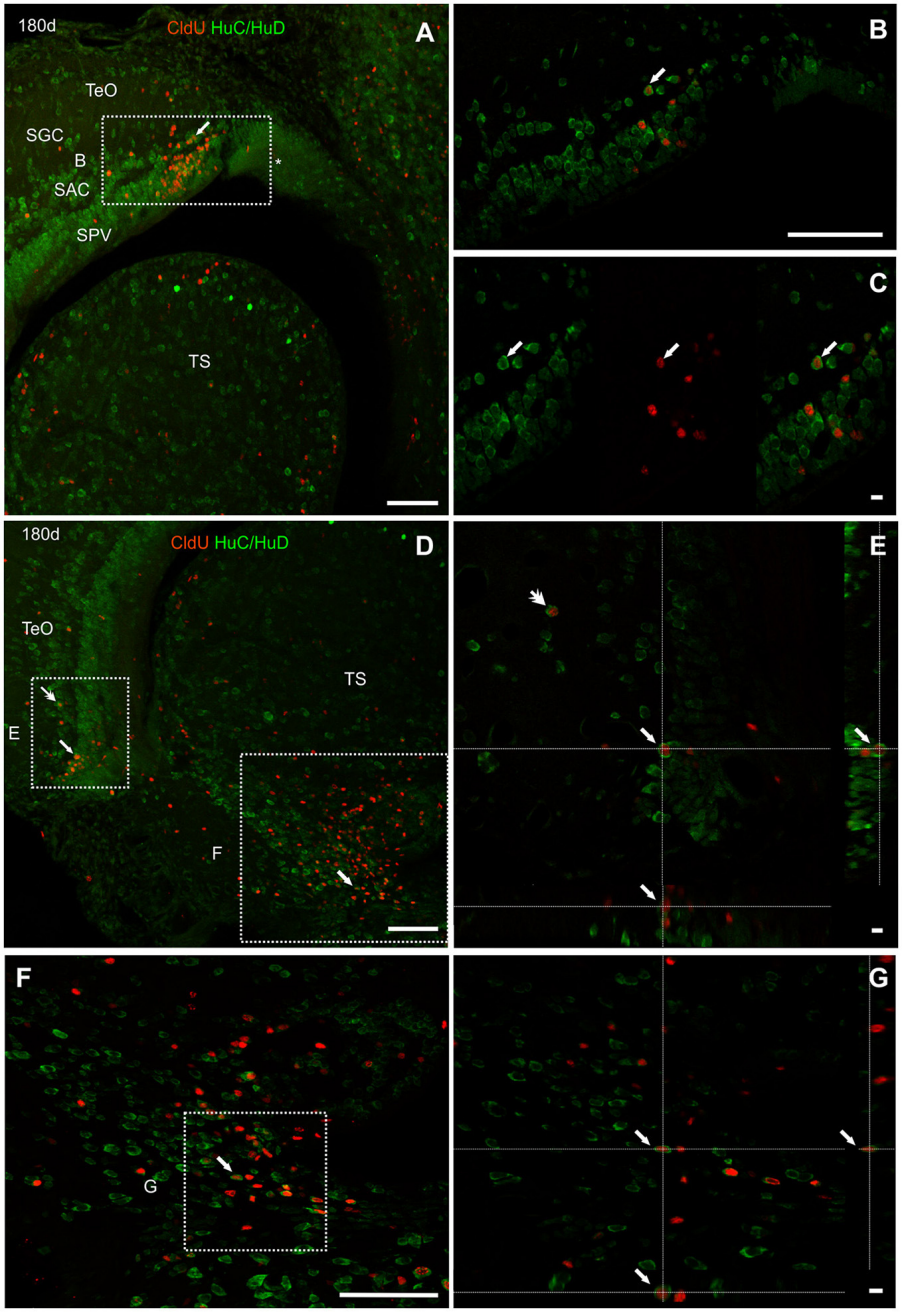
Neuronal differentiation in the intermediate region of the optic tectum and torus semicircularis at 90 and 180 days after four daily injections of CldU demonstrated by co-localization with HuC/HuD. **(A,B)** Low power microphotographs at the dorsal-medial and ventral-lateral border of the tectum opticum (TeO) and dorsal region of the torus semicircularis **(TS)**, rostral to the caudal pole. The dotted rectangles indicate the location of three regions of interest observed at higher magnification in **(B,E,F)**, respectively. Double labeling of tectal cells **(B,C,E)** and toral newborn cells **(F,G)** was confirmed by the overlay of each channel **(C)** and *xy* and *zy* orthogonal projections a stacks. Scale bars: **(A**,**B**,**D**,**F)** 20 μm; (**B**, Inset in **A** and **D**) 100 μm; **(C**,**E**,**G)** 5 μm.

One hundred eighty days after CldU administration, derived cells were mainly grouped at the SPV and SAC layers at about 100 μm from the rostral portion of the dorsal-medial region of the TeO proliferation zone (asterisk in Figure 13A). Some of these cells expressed HuC/HuD (arrows in Figures 13A–C). Conversely, most of CldU labeled were not displaced from the ventro-lateral region of the tectal proliferation zone (arrow in Figure 13D); few others were located quite far from it (double arrows in Figures 13D,E). Some of these long term CldU label retaining cells also expressed HuC/HuD (Figure 13E).

In the ventral region of the TS, long term CldU labeled cells were distributed in and extended zone with numerous double CldU-HuC/HuD labeled cells (Figures 13D,F,G).

##### Electrosensory lateral line lobe

**Figure 14:**
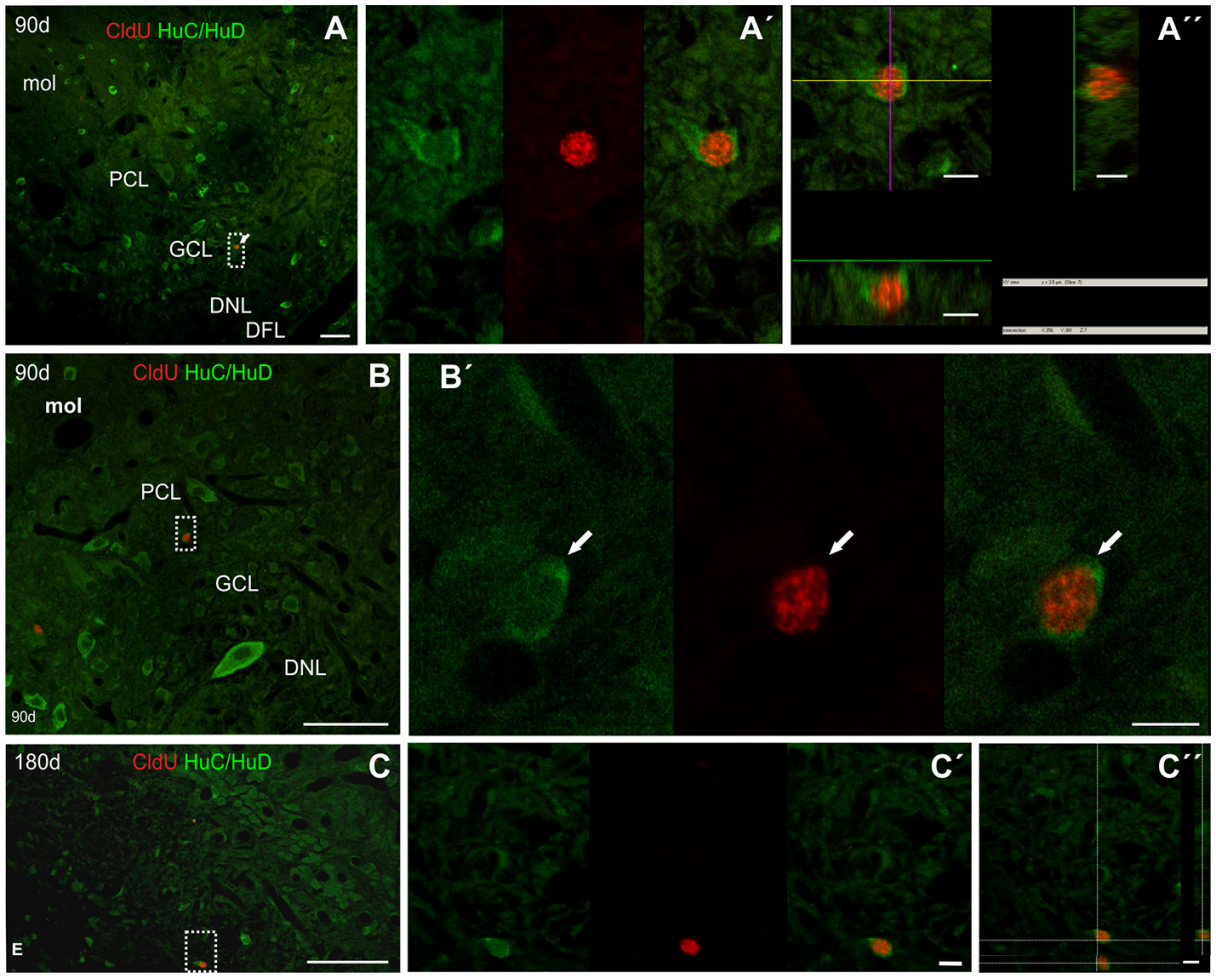
Neuronal differentiation in the caudal pole of the electrosensory lateral line lobe at 90 and 180 days after four daily injections of CldU demonstrated by co-localization with HuC/HuD. **(A–C)** Low power microphotographs of frontal sections at the lateral-caudal border of the electrosensory lateral line lobe. The dotted rectangles indicate the location of double labeled CldU-HuC/HuD newborn cells as evidenced by the overlays of sequentially acquired images **(A**′**–C**′**)** and orthogonal projections of stacks **(A**′′**,C**′′**)**. DNL, deep neuropile layer of ELL; DFL, deep fiber layer of ELL; GCL, granular cell layer of ELL; PCL, pyramidal cell layer of ELL; mol, molecular layer of ELL. Scale bars: **(A–C)** 50 μm; **(A**′**,A**′′**,B**′**,C**′**,C**′′**)** 5 μm.

Unlike other brain regions, very scarce CldU long-term label retaining cells were found at the caudal region of the ELL 90 (Figures 14A,B) or 180 (Figure 14C) days after CldU administration. Some of these cells with a thin fusiform to ovoid cytoplasmic halo were located at the GCL, near the boundaries with adjacent layers, also expressing HuC/HuD (Figures 14 A′,A′′,B′,C′,C′′).

Considering the quantitative data from all the analyzed brain regions (OB, TeO, TS and CCb), 50% of long term CldU retaining new born cells expressed HuC/HuD 90 days after CldU administration (range: TS: 33,33—CCb: 57,14). The fraction of double labeled cells increased up to almost 80% in the TS and TeO after 180 day chase (Table 1).

#### Neuronal differentiation in the CCb evidenced by CldU and neurobiotin

**Figure 15:**
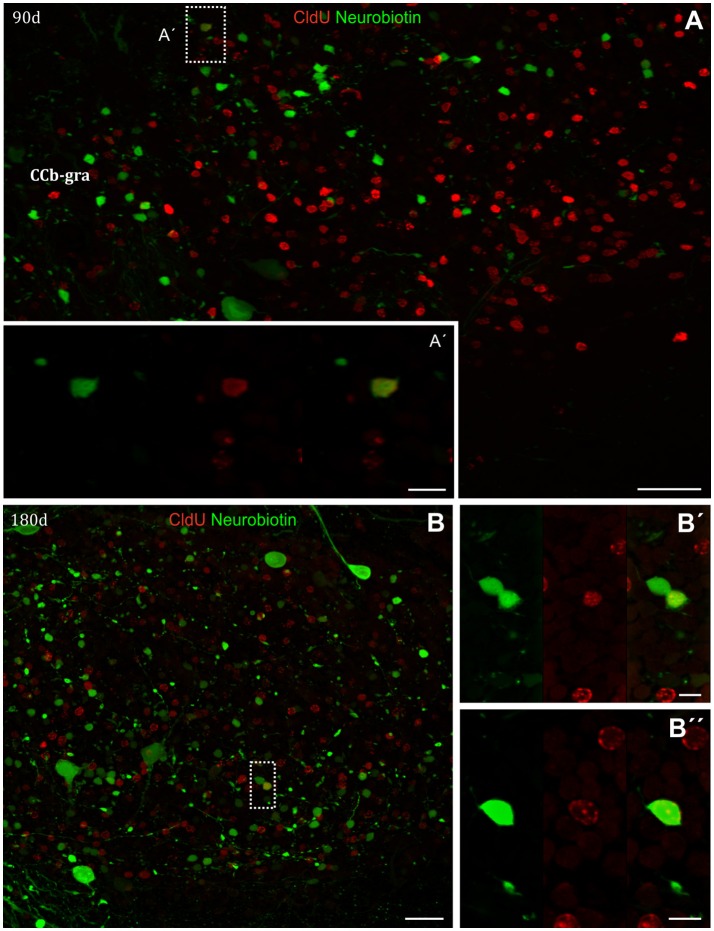
Neuronal differentiation in the corpus cerebelli at 90 and 180 days after four daily injections of CldU demonstrated by co-localization retrogradely transported Neurobiotin. **(A,B)** Low power microphotograph of frontal sections at the corpus cerebelli (CCb). The dotted rectangles indicate the location of double labeled newborn cells nearby the boundary between molecular (CCb-mol) and granular (CCb-gra) layer of the CCb **(A)**, or within CCb-gra **(B)**. The double labeling was confirmed at higher magnification by the microphotographs of each channel and the overlays **(A**′**,B**′**,B**′′**)** as well as by the 3-D visualization as shown in the Supplementary Video 2. Scale bars: **(A)** = 20 μm; (**B**, Inset in **A**) = 100 μm; **(A**′**,B**′**,B**′′**)** = 5 μm.

Repetitive CldU administration allowed the long term retention of CldU in numerous cells at the CCb-gra, both 90 (Figure 15A) and 180 days (Figure 15B) after thymidine analog administration. These derived cells probably correspond to granule cells, both because of their location, as well as the shape and size of their nuclei. Cortical application of Neurobiotin labeled many cells with small round, ovoid, or polygonal soma, at the CCb-gra at both chase durations, even far from the site of tracer application. Note that the site of injection is not visible in the topographic images of Figures 15A,B, and thus is located more than 200 μm from the soma of the Neurobiotin labeled cells. This indicates that the tracer was incorporated into the axons or end terminals of the labeled cells at the CCb-mol and retrogradely transported up to the cell's soma and perisomatic dendrites at the CCb-gra. This, as well as the known histoarchitectural organization of the Cb, further supports the identification of these cells as granule cells. Many long term CldU retaining cells were labeled with Neurobiotin (Figures 15A′,B′,B″, Supplementary Video 2), indicating that the newborn cells acquired the mature granular morphology in 90 days. Also indicates that these newborn granule cells very probably were incorporated into the local cerebellar circuit and might be already functional. Conversely, Purkinje cells were also labeled with Neurobiotin but not CldU.

The fraction of CldU CCb-gra labeled cells that were retrogradely labeled represented almost 1% and more than 9% after chase durations of 90 and 180 days, respectively (Table 1).

## Discussion

Adult neurogenesis, whether involved in protracted postnatal development of parts of the brain, the persistent addition of new neurons (constitutive neurogenesis), cellular turnover, or the regeneration after injury (Grandel and Brand, 2013) is a widespread process in the animal kingdom, from the most primitive species in which the nervous system first evolved to diverse vertebrate radiations.

Though adult cell proliferation was studied in teleost (Rahmann, 1968) soon after the discovery of adult neurogenesis in mammals by Altman (1962), only few species of the numerous teleostean radiation have been studied. Even fewer are the teleost species in which adult neurogenesis was demonstrated. Remarkably, proliferating cells give place to newborn neurons in a period as short as 24 h after BrdU administration in several brain regions (OB, TEL, TO, TL, and Cb) of *Austrolebias* (Fernández et al., 2011; Rosillo et al., 2016), probably related to the short duration of their life cycle. In all other studied teleost species, longer chases are necessary for the demonstration of neurogenesis: 3 days in the dorsal telencephalon of *D. rerio* (Adolf et al., 2006), 7 days in the TEL, TeO, and Cb of *N. furzeri* (Terzibasi et al., 2012), 15 days in the OB and TEL of *D. rerio* (Adolf et al., 2006). Almost 50% of the total amount of proliferating cells differentiates into neurons at 270–744 days chases in *D. rerio* (Zupanc et al., 2005; Hinsch and Zupanc, 2007) though a great variation among brain regions exists. A high and variable proportion of adult brain cells expressing HuC/HuD in *O. mossambicus* was demonstrated 100 days after BrdU administration (Teles et al., 2012).

*G. omarorum* brain develops rostro-caudally; as morphogenesis develops, the widespread distribution of larval brain proliferating cells is progressively reduced but persist in several ventricular and extraventricular proliferation zones up to adulthood (Iribarne and Castelló, 2014; Olivera-Pasilio et al., 2014). In the present work we deepened the study of the migration process (particularly at the rostral telencephalon and OB), and demonstrate the differentiation into the neuronal phenotype of newborn cells generated in telencephalic, mesencephalic, and rhombencephalic proliferation zones.

### Adult *G. omarorum* brain proliferation zones are the source of long-range migratory streams

According to the chase and the brain region, newborn cells were found close to the proliferation zones from where they originate or at increasing distances from their boundaries. At 7–180 days survivals after CldU administration we evidenced wide-ranging migrating cells with thin and elongated nuclei (usually intensely stained with CldU) surrounded by thin elongated cellular process of diverse lengths, typical characteristics of migrating neuroblasts. Other cells had round to ovoid nuclei, with granular or diffuse CldU staining, indicative of cells that already reached their final location.

No proliferation zone was found at the rostral region of *G. omarorum* OB. Despite that, the OB was progressively populated by CldU labeled cells at chase durations from 7 to 180 days.

Considering that the nearest proliferation zone was found at the ventricular lining of the rostralmost region of the telencephalic ventricle (adjacent to the caudal portion of OB and the rostral portion of the subpallium), we inferred that this is the main source of OB newborn cells. Taking into account the location of CldU labeled nuclei at the three telencephalic levels studied as a function of chase duration, we propose a three-step migration process. First, a medial-lateral migration from the proliferation 1b to adjacent regions of the subpallium and caudal OB; second, a caudal-rostral migration along the ICL and the dorsal-medial zone of the caudal and rostral OB; finally, a radial migration to populate other layers of caudal and rostral levels of OB. This resembles the rostral migratory stream from the telencephalic ventricle to the OB described in mammals.

Even though the rostral migratory stream was first demonstrated in mammals (Altman, 1969), a similar mechanism of cell proliferation at the wall of the telencephalic ventricle and collective cellular migration was also demonstrated in birds (Goldman and Nottebohm, 1983; Nottebohm, 2002; Barnea and Pravosudov, 2011), and only recently identified in *D. rerio* (Adolf et al., 2006; Grandel et al., 2006; Pellegrini et al., 2007), and supported by *ex vivo* imaging by Kishimoto et al. (2011). The generality of this process among teleost was suggested by Olivera-Pasilio et al. (2014) and supported by Lasserre (2014) in the phylogenetically close teleost *G. omarorum* and by Rosillo et al. (2016) in *Austrolebias charrua*, a Cyprinodontiforme phylogenetically very distant from the former teleosts. Consistently, here we showed that the ICL and dorsal-medial zone of the OB of *G. omarorum* contains rostral-caudally oriented cellular processes that are reactive to DCX. This microtubule associated protein is expressed by migrating neuroblasts during development (Francis et al., 1999) up to adulthood in the telencephalon of mammals (Gleeson et al., 1999; Lim et al., 2008; including the rostral migratory stream on humans, Wang et al., 2011), and birds (Boseret et al., 1997). In our knowledge DCX was only demonstrated in the telencephalon and mesencephalon the teleost, *Nothobranchius furzeri* (Terzibasi et al., 2012).

Nevertheless, other authors demonstrated local proliferation zones at superficial layers of the OB in *C. auratus, Barbus meridionalis, Cyprinus carpio*, and *Salmo gardneri* (Alonso et al., 1989), *Oreochromis mossambicus* (Teles et al., 2012), *A. leptorhynchus* (Zupanc and Horschke, 1995), and *D. rerio* (Zupanc et al., 2005) and local migration between layers of the OB.

Our results also support a simultaneous process of radial migration of newborn cells from the proliferation zone nearby rostral subpallium and caudal OB to the surrounding subpallial cell masses, as indicated by the distribution of CldU labeled nuclei (Supplementary Figure 3), similar to the findings of Grandel et al. (2006) in *D. rerio*.

Other outstanding proliferation and migration processes occurred in the Cb of adult *G. omarorum* as newborn cells migrated along relatively large distances in all cerebellar divisions (CCb, VCb, and EG) as they relocate from one cerebellar layer to another. There, the migration process involved an almost complete shift of newborn cells between cerebellar layers in a period of 30 days after CldU administration, as previously shown in the Cb of this (Olivera-Pasilio, 2014) and other teleost species (*A. leptorhynchus*: Zupanc et al., 1996; *D rerio*: Zupanc et al., 2005; Kaslin et al., 2009; *C. auratus*: Delgado and Schmachtenberg, 2011; *O. mossambicus*: Teles et al., 2012; and *M. rume*: Radmilovich et al., 2016). According to the distribution of DCX cellular process in the CCb of *G. omarorum*, the migration process of new born cells may involve displacements in the medial-lateral direction along the CCb-mol (as shown by Kaslin et al., 2009), as well as in the rostral-caudal direction in the ganglionic layer, not described previously.

Shorter range or less numerous migration processes were found in other mesencephalic (TeO and OT) and rhombencephalic brain regions (ELL) involved in multimodal and electrosensory information processing. The migration process of newborn cells in the TeO of *G. omarorum* appeared to occur mainly from the caudal pole of the tectal proliferation zone toward almost all tectal layers as well as from the dorso-medial edge of more rostral zones of the tectal proliferation zone. This involves both the addition of newborn cells to the dorso-medial edge to the tectal proliferation zone, and the displacement of older cells to adjacent caudal parts of the TeO in the ventro-lateral direction as demonstrated in *O. latipes* (Alunni et al., 2010), *C. auratus* (Raymond and Easter, 1983) and *D. rerio* (Ito et al., 2010), and *N. furzeri* (Terzibasi et al., 2012). Consistently, a net of DCX process lies beneath *G. omarorum* dorsal-medial tectal proliferation zone.

### Neurogenic potential of adult *G. omarorum* brain proliferation zones

After long term chases, the nuclei of most derived cells lost the typical appearance of migrating cells as they reached their target brain regions. Nuclei acquired a rounded shape with heterogeneous distribution of loosely and densely packed chromatin, frequently showing a carriage wheel arrangement of compacted chromatin, as evidenced by thymidine analog labeling. This indicates that these cells are already in the process of cell differentiation that was confirmed by the demonstration of the expression of early or mature neuronal markers, or the retrograde transport of Neurobiotin by long term CldU label retaining cells.

Even though at short chases (1 or 7 days) some derived cells had the aspect of migrating neuroblasts; most of these cells do not express the early neuronal marker DCX, with the exception of few cells nearby the ventral zone of the telencephalic proliferation zone 1b, similar to *N. furzeri* (Terzibasi et al., 2012). Conversely, 7 days were sufficient for the expression of HuC/HuD by CldU label retaining cells nearby the subpallial proliferation zone, indicating their differentiation into the neuronal phenotype. Some newborn cells nearby the ventricular surface of the subpallial proliferation zone express DCX at a chase duration of 30 days, a slightly longer time interval than observed in mammal hippocampus (Kempermann et al., 2008) and *N. furzeri* (Terzibasi et al., 2012). This and their morphology indicate that they correspond to migrating neuroblasts. At this chase, other newborn neurons expressing HuC/HuD were located laterally to the subpallial proliferation zone, or rostrally, at the ICL of caudal OB. Longer chases were required for newborn neurons expressing HuC/HuD to reach the ICL of the rostral OB, supporting a long-range migration from the telencephalic ventricle. Strikingly, CldU-HuC/HuD double labeled cells reached more than 50% of newborn migrated cells 90 days after CldU administration. To our knowledge, this has not been reported before, since most of previous studies combining HuC/HuD and thymidine analog retention to demonstrate the differentiation into the neuronal phenotype in the OB failed, either to identify cells expressing HuC/HuD (Zupanc et al., 2005) or the co-localization of HuC/HuD and the thymidine analog (Adolf et al., 2006), or find very few double labeled cells (Grandel et al., 2006). Only the quantitative results of Hinsch and Zupanc (2007) support a 30% fraction of BrdU labeled cells also expressing HuC/HuD after chases between 446 and 656 days.

On the other hand, Alonso et al. (1989) not only found a proliferation zone in the wall of the telencephalic ventricle or ventricular recess but also another at the outer limiting glial membrane and the olfactory nerve fiber layer of *C. auratus, B. meridionalis, C. carpio*, and *Salmo gairdneri*. Superficial proliferation zones at the primary olfactory fiber layer and/or the glomerular layer of the OB were also found in other teleost (*O. mossambicus*: Teles et al., 2012; *A. leptorhynchus*: Zupanc and Horschke, 1995; *D. rerio*: Zupanc et al., 2005) that give rise to newborn cells that migrate toward the inner cell layers. Only in *O. mossambicus* it was shown a high proportion of newborn cells originated from the superficial proliferation zone of the OB expressing HuC/HuD (Teles et al., 2012). All these findings suggest that there are marked interspecific differences in the site of generation, the paths of migration and fate of newborn cells.

A step forward in the process of neuronal differentiation was evidenced by the expression of a more cell specific neuronal marker: TH, the key step-limiting enzyme of the catecholamine synthesis pathway. TH first co-localize with CldU at a chase of 90 days in newborn neurons located within or nearby the subpallial proliferation zone. At 180 chase duration (similar to CldU-HuC/HuD double labeled cells), CldU-TH cells were found further away from the proliferation zones, both in the medial lateral direction (reaching the MOTF and Vc) and in the rostral caudal direction (reaching the ICL of caudal and rostral OB). This is coincident with the results of Grandel et al. (2006) and Adolf et al. (2006) in *D. rerio*, though in *G. omarorum* it was not a rare finding. Conversely, according to our quantitative data, newborn catecholaminergic neurons reached 0,5% of migrated newborn neurons of the subpallium 90 days after CldU administration, a figure that was duplicated and triplicated at caudal and intermediate regions of the OB at the longest chase studied (180 days). The population of newborn catecholaminergic neurons was not homogeneous, but showed morphological characteristics corresponding to different steps in the process of cell maturation (from migrating neuroblasts to multipolar granular cells). According to the morphology and location, double labeled CldU-TH newborn cells of the OB of *G. omarorum* correspond to granule cells of the ICL as shown in other teleosts (*Dicentrarchus labrax*: Batten et al., 1993; *D. rerio*: Byrd and Brunjes, 1995; and *Solea senegalensis*: Rodríguez-Gómez et al., 2000). However, this is not coincident with the distribution of catecholaminergic neurons in the OB of *A. leptorhynchus* (Sas et al., 1990), *D. rerio* (Adolf et al., 2006), and *D. labrax* (Sébert et al., 2008) as in these species TH positive cells are almost absent in the ICL and more abundant in outer layers.

According to our results and, it takes 30 days for newborn cells of the CCb-gra to express the early neuronal marker HuC/HuD, indicating their differentiation into granule cells. This is similar to *D. rerio* but either much longer (Grandel et al., 2006) or shorter (Kaslin et al., 2009) chase durations were reported, or even absence of double labeling of CCb-gra newborn cells even at very long chase durations (Zupanc et al., 2005). Conversely, in *Austrolebias*, a 1 day chase is enough for co-localization of BrdU and HuC/HuD (Fernández et al., 2011). These differences may be due to variations in the sensitivity of immunohistochemical procedures, or in the process of cell differentiation because of differences in the durations of life span as argued before.

More than half of newborn CCb-gra cells express HuC/HuD in *G. omarorum* at 90 day chase duration, a value that amply surpass the fraction of CCb new born cells in *D. rerio* at 446–656 day chases (2,4%, according to the numbers of BrdU and BrdU-HuC/HuD double labeled cells reported by Hinsch and Zupanc, 2007).

HuC/HuD is also expressed by tectal and toral newborn neurons but requiring longer chases (90–180 days). In *D. rerio*, double labeled BrdU-HuC/HuD newborn neurons were found nearby the proliferation zone of the TeO (Zupanc et al., 2005; Grandel et al., 2006). The cortical organization of the TeO and the distribution of newborn neurons in almost all its width, suggest the coordinated migration of cohorts of newborn cells of various phenotypes. The fraction of TeO double labeled cells here found in adult *G. omarorum* also largely exceeds the values in *D. rerio* according to the quantitative results reported in Hinsch and Zupanc (2007).

We also found newborn double labeled CldU-HuC/HuD neurons in the ELL, though they show weak HuC/HuD immunoreactivity. To our knowledge, the generation of new neurons (by demonstration of HuC/HuD expression in thymidine analog retaining cells) has not been reported neither in the TS nor the ELL of teleosts. It is interesting to note that all these brain regions are involved in sensory information processing, particularly of electrosensory information.

To identify the neuronal phenotype of newborn neurons, we draw upon the combination of repetitive thymidine analog labeling and “*in vivo*” neuronal tracing to retrogradely label cerebellar and ELL granule cells. This approach rendered a high proportion of granule cells retrogradely labeled, even at distances of more than 200 microns of the site of Neurobiotin application, as well as a high proportion of long term CldU retaining granule cells. These facts probably favored the identification of several double labeled CldU-Neurobiotin granule cells, indicating that these cells already acquired a mature phenotype with axonal projections to the CCb-mol. Similar, though quantitatively much less frequent results were obtained in *A. leptorhynchu*s (Zupanc et al., 1996) and *D. rerio* (Zupanc et al., 2005) by “*ex vivo*” retrograde labeling of granule cells with dextran-fluorescein. We did not identify double labeling of any of the other Cb cell types. Finally, unlike the CCb, we did not observe double labeled CldU-Neurobiotin granule cells in the ELL, even though Neurobiotin application to the lateral line nerve rendered abundant trans-synaptic labeled granule cells (data not shown).

In summary, the results of this work confirm the spatial distribution of adult *G. omarorum* brain proliferation zones, and the migration paths of newborn cells from the proliferation zones to their final locations, particularly at the rostral telencephalon, TeO, TS, and CCb. Our results are compatible with a rostral migratory stream of newborn cells from a proliferation zone at the rostralmost end of the telencephalic ventricle toward the rostral and caudal OB. We also demonstrate widespread and relatively frequent neurogenesis in the telencephalon (subpallium and OB), mesencephalon (TeO and TS), and rhombencephalon (CCb and ELL) of adult *G. omarorum*. These findings contribute to support the widespread distribution of brain proliferation zones and their neurogenic capacity in *G. omarorum*, an animal model suitable to assess the functional significance, as well as comparative analysis of adult neurogenesis. Our results also contribute to support the phylogenetically conserved feature of adult neurogenesis. Considering the rough similarity in distribution of brain proliferation zones among teleost species studied up to date, the differences in the neurogenic capacity between the same regions among teleosts suggest differences in the intrinsic/extrinsic factors modulating both cell proliferation and neurogenesis, which is the topic of ongoing research.

## Author contributions

MC, ML, and VO made substantial contributions to the conception or design of the work; and/or the acquisition, analysis, and interpretation of data for the work; MC and VO drafted the work. Final approval of the version to be published was done by VO and MC.

### Conflict of interest statement

The authors declare that the research was conducted in the absence of any commercial or financial relationships that could be construed as a potential conflict of interest.

## Supplementary material

Supplementary Video 1 Newborn neuron in the olfactory bulb of adult *G. omarorum*. The video corresponds to an animation obtained with Fluoview from a stack of confocal images obtained from a frontal section of the caudal OB processed for the immunohistochemical detection of tyrosine hydroxilase (green) and CldU (red). The stack consisted in 13 confocal planes, acquired in sequential mode, every 1 μm. Note in the center of the animation a double labeled newborn neuron, corresponding to the cell shown in Figure 10C, as well as other single labeled TH+ or CldU+ cells. Online available at: https://figshare.com/s/0fab179fd0e1e6356371.

Supplementary Video 2 Newborn neuron in the corpus cerebelli of adult *G. omarorum*. The video corresponds to an animation obtained with Fluoview from a stack of confocal images obtained from a frontal section of the corpus cerebelli (CCb) processed for the immunohistochemical detection CldU (red) and histochemical demonstration of Neurobiotin (green). Note in the right of the animation a double labeled newborn neuron, corresponding to the cell shown in Figure 15B, as well as other single labeled Neurobiotin+ or CldU+ cells. Online available at: https://figshare.com/s/af3f18ae8aea51d80b27.

## References

[B1] Adolf B. Chapouton P. Lam C. S. Topp S. Tannhäuser B. Strähle U.. (2006). Conserved and acquired features of adult neurogenesis in the zebrafish telencephalon. Dev. Biol. 295, 278–293. 10.1016/j.ydbio.2006.03.023 16828638

[B2] Albert J. Lannoo M. Yuri T. (1998). Testing hypotheses of neural evolution in gymotiform electric fishes using phylogenetic character data. Evolution 52, 1760–1860. 28565314 10.1111/j.1558-5646.1998.tb02255.x

[B3] Alonso J. R. Lara J. Vecino E. Cove-as R. Aijón J. (1989). Cell proliferation in the olfactory bulb of adult freshwater teleosts. J. Anat. 163, 155–163. 2606770 PMC1256525

[B4] Altman J. (1962). Are new neurons formed in the brains of adult mammals? Science 135, 1127–1128. 10.1126/science.135.3509.1127 13860748

[B5] Altman J. (1963). Autoradiographic investigation of cell proliferation in the brains of rats and cats. Anat. Rec. 145, 573–591. 10.1002/ar.1091450409 14012334

[B6] Altman J. (1969). Autoradiographic and histological studies of postnatal neurogenesis. IV. Cell proliferation and migration in the anterior forebrain, with special reference to persisting neurogenesis in the olfactory bulb. J. Comp. Neurol. 137, 433–457. 10.1002/cne.901370404 5361244

[B7] Altman J. (2011). The discovery of adult mammalian neurogenesis, in Neurogenesis in the Adult Brain I, eds Seki T. Sawamoto K. Parent J. M. Alvarez-Buylla A. (Tokyo: Springer Japan), 3–46.

[B8] Altman J. Das G. (1965). Autoradiographic and histological evidence of postnatal hippocampal neurogenesis in rats. J. Comp. Neurol. 124, 319–335. 10.1002/cne.901240303 5861717

[B9] Altman J. Das G. D. (1966). Autoradiographic and histological studies of postnatal neurogenesis. I. A longitudinal investigation of the kinetics, migration and transformation of cells incorporating tritiated thymidine in neonate rats, with special reference to postnatal neurogenesis. J. Comp. Neurol. 126, 337–389. 5937257 10.1002/cne.901260302

[B10] Alunni A. Bally-Cuif L. (2016). A comparative view of regenerative neurogenesis in vertebrates. Development 1, 741–753. 10.1242/dev.122796 PMC4813331 26932669

[B11] Alunni A. Hermel J.-M. Heuzé A. Bourrat F. Jamen F. Joly J.-S. (2010). Evidence for neural stem cells in the medaka optic tectum proliferation zones. Dev. Neurobiol. 70, 693–713. 10.1002/dneu.20799 20506557

[B12] Balon E. K. (1975). Terminology of intervals in fish development. J. Fish. Res. Board Can. 32, 1663–1670. 10.1139/f75-196

[B13] Barbieri G. Cruz M. (1983). Growth and first sexual maturation size of *Gymnotus carapo* (Linnaeus, 1758) in the Lobo reservoir (state of Sao Paulo, Brazil) (pisces, gymnotidae). Rev. Hydrobiol. Trop. 16, 195–201.

[B14] Barker J. M. Boonstra R. Wojtowicz J. M. (2011). From pattern to purpose: how comparative studies contribute to understanding the function of adult neurogenesis. Eur. J. Neurosci. 34, 963–977. 10.1111/j.1460-9568.2011.07823.x 21929628

[B15] Barnea A. Pravosudov V. (2011). Birds as a model to study adult neurogenesis: bridging evolutionary, comparative and neuroethological approaches. Eur. J. Neurosci. 34, 884–907. 10.1111/j.1460-9568.2011.07851.x 21929623 PMC3177424

[B16] Batten T. F. C. Berry P. A. Maqbool A. Moons L. Vandesande F. (1993). Immunolocalization of catecholamine enzymes, serotonin, dopamine and L-dopa in the brain of *Dicentrarchus labrax* (Teleostei). Brain Res. Bull. 31, 233–252. 10.1016/0361-9230(93)90214-V 8098256

[B17] Bell C. C. (2002). Evolution of cerebellum-like structures. Brain. Behav. Evol. 59, 312–326. 10.1159/000063567 12207086

[B18] Bennett M. V. L. (1971). Electric organs, in Fish Physiology, eds Hoar W. S. Randall D. J. (London: Academic Press), 347–491.

[B19] Boseret G. F. Ball G. F. Balthazart J. (1997). The microtubule-associated protein doublecortin is broadly expressed in the telencephalon of adult canaries. J. Chem. Neuroanat. 33, 140–154. 10.1016/j.jchemneu.2007.02.002 17367992 PMC2040224

[B20] Byrd C. A. Brunjes P. C. (1995). Organization of the olfactory system in the adult zebrafish: histological, immunohistochemical, and quantitative analysis. J. Comp. Neurol. 358, 247–259. 10.1002/cne.903580207 7560285

[B21] Cayre M. Malaterre J. Scotto-Lomassese S. Strambi C. Strambi A. (2002). The common properties of neurogenesis in the adult brain: from invertebrates to vertebrates. Comp. Biochem. Physiol. B Biochem. Mol. Biol. 132, 1–15. 10.1016/S1096-4959(01)00525-5 11997205

[B22] Corrêa S. A. Corrêa F. M. Hoffmann A. (1998). Stereotaxic atlas of the telencephalon of the weakly electric fish *Gymnotus carapo*. J. Neurosci. Methods 84, 93–100. 10.1016/S0165-0270(98)00098-3 9821639

[B23] Delgado L. M. Schmachtenberg O. (2011). Neurogenesis in the adult goldfish cerebellum. Anat. Rec. 294, 11–15. 10.1002/ar.21291 21157912

[B24] Dunlap K. D. Silva A. C. Chung M. (2011). Environmental complexity, seasonality and brain cell proliferation in a weakly electric fish, *Brachyhypopomus gauderio*. J. Exp. Biol. 214, 794–805. 10.1242/jeb.051037 21307066 PMC3036548

[B25] Evans H. M. (1940). Brain and Body Fish. A Study of Brain Pattern in Relation to Hunting and Feeding in Fish. Norwich: London and Norwich Press.

[B26] Fernández A. S. Rosillo J. C. Casanova G. Olivera-Bravo S. (2011). Proliferation zones in the brain of adult fish *Austrolebias* (Cyprinodontiform: Rivulidae): a comparative study. Neuroscience 189, 12–24. 10.1016/j.neuroscience.2011.05.063 21664435

[B27] Finger T. E. (1978). Efferent neurons of the teleost cerebellum. Brain Res. 153, 608–614. 10.1016/0006-8993(78)90346-3 81089

[B28] Francis F. Koulakoff A. Boucher D. Chafey P. Schaar B. Vinet M. C.. (1999). Doublecortin is a developmentally regulated, microtubule-associated protein expressed in migrating and differentiating neurons. Neuron 23, 247–256. 10.1016/S0896-6273(00)80777-1 10399932

[B29] Galliot B. Quiquand M. (2011). A two-step process in the emergence of neurogenesis. Eur. J Neurosci. 34, 847–862. 10.1111/j.1460-9568.2011.07829.x 21929620

[B30] Gleeson J. G. Lin P. T. Flanagan L. A. Walsh C. A. (1999). Doublecortin is a microtubule-associated protein and is expressed widely by migrating neurons. Neuron 23, 257–271. 10.1016/S0896-6273(00)80778-3 10399933

[B31] Goldman S. A. Nottebohm F. (1983). Neuronal production, migration, and differentiation in a vocal control nucleus of the adult female canary brain. Proc. Natl. Acad. Sci. U.S.A. 80, 2390–2394. 10.1073/pnas.80.8.2390 6572982 PMC393826

[B32] Grandel H. Brand M. (2013). Comparative aspects of adult neural stem cell activity in vertebrates. Dev. Genes Evol. 223, 131–147. 10.1007/s00427-012-0425-5 23179636

[B33] Grandel H. Kaslin J. Ganz J. Wenzel I. Brand M. (2006). Neural stem cells and neurogenesis in the adult zebrafish brain: origin, proliferation dynamics, migration and cell fate. Dev. Biol. 295, 263–277. 10.1016/j.ydbio.2006.03.040 16682018

[B34] Gross C. G. (2000). Neurogenesis in the adult brain: death of a dogma. Nat. Rev. Neurosci. 1, 67–73. 10.1038/35036235 11252770

[B35] Haugedé-Carré F. Kirschbaum F. Szabo T. (1977). On the development of the gigantocerebellum in the mormyrid fish *Pollimyrus (Marcusenius) isidori*. Neurosci. Lett. 6, 209–213. 10.1016/0304-3940(77)90020-9 19605054

[B36] Haugedé-Carré F. Szabo T. Kirschbaum F. (1979). Development of the gigantocerebellum of the weakly electric fish *Pollimyrus*. J. Physiol. Paris 75, 381–395. 512971

[B37] Hinsch K. Zupanc G. K. H. (2007). Generation and long-term persistence of new neurons in the adult zebrafish brain: a quantitative analysis. Neuroscience 146, 679–696. 10.1016/j.neuroscience.2007.01.071 17395385

[B38] Hodos W. Butler B. (1997). Evolution of sensory pathways in vertebrates. Brain Behav. Evol. 50, 189–197. 10.1159/000113333 9310194

[B39] Iribarne L. Castelló M. E. (2014). Postnatal cell proliferation in the brain of the weakly electric fish *Gymnotus omarorum*. J. Physiol. Paris 108, 47–60. 10.1016/j.jphysparis.2014.05.001 24844821

[B40] Ito H. Ishikawa Y. Yoshimoto M. Yamamoto N. (2007). Diversity of brain morphology in teleosts: brain and ecological niche. Brain. Behav. Evol. 69, 76–86. 10.1159/000095196 17230015

[B41] Ito Y. Tanaka H. Okamoto H. Ohshima T. (2010). Characterization of neural stem cells and their progeny in the adult zebrafish optic tectum. Dev. Biol. 342, 26–38. 10.1016/j.ydbio.2010.03.008 20346355

[B42] Jerisson H. J. (1973). Evolution of the Brain and Intelligence. London: Academic Press, Inc. (London) Ltd.

[B43] Kaplan M. S. Hinds J. (1977). Neurogenesis in the adult rat: electron microscopic analysis of light radioautographs. Science 197, 1092–1094. 10.1126/science.887941 887941

[B44] Kaslin J. Ganz J. Brand M. (2008). Proliferation, neurogenesis and regeneration in the non-mammalian vertebrate brain. Philos. Trans. R. Soc. Lond. B Biol. Sci. 363, 101–122. 10.1098/rstb.2006.2015 17282988 PMC2605489

[B45] Kaslin J. Ganz J. Geffarth M. (2009). Stem cells in the adult zebrafish cerebellum: initiation and maintenance of a novel stem cell niche. J. Neurosci. 29, 6142–6153. 10.1523/JNEUROSCI.0072-09.2009 19439592 PMC6665484

[B46] Kempermann G. Song H. Gage F. H. (2008). Neurogenesis in the adult hippocampus, in Adult Neurogenesis, eds Gage F. H. Kempermann G. Song H. (New York, NY: Cold Spring Harbor Laboratory Press), 159–174. 10.1101/087969784.52.159

[B47] Kermen F. Franco L. M. Wyatt C. Yaksi E. (2013). Neural circuits mediating olfactory-driven behavior in fish. Front. Neural Circuits 7:62. 10.3389/fncir.2013.00062 23596397 PMC3622886

[B48] Kishimoto N. Alfaro-Cervello C. Shimizu K. Asakawa K. Urasaki A. Nonaka S.. (2011). Migration of neuronal precursors from the telencephalic ventricular zone into the olfactory bulb in adult zebrafish. J. Comp. Neurol. 519, 3549–3565. 10.1002/cne.22722 21800305

[B49] Kotrschal K. Van Staaden M. Huber R. (1998). Fish brains: evolution and environmental relationships. Rev. Fish Biol. Fish. 8, 373–408. 10.1023/A:1008839605380

[B50] Lannoo M. J. Vischer H. A. Maler L. (1990). Development of the electrosensory nervous system of Eigenmannia (gymnotiformes): II. The electrosensory lateral line lobe, midbrain, and cerebellum. J. Comp. Neurol. 294, 37–58. 10.1002/cne.902940105 2324333

[B51] Lasserre M. (2014). Evaluación de la Capacidad Neurogénica Adulta en el Telencéfalo de Gymnotus omarorum. Dissertation/undergraduate thesis, Universidad de la República, Montevideo.

[B52] Leyhausen C. Kirschbaum F. Szabo T. Erdelen M. (1987). Differential growth in the brain of the weakly electric fish, *Apteronotus leptorhynchus* (Gymnotiformes), during ontogenesis (Part 1 of 2). Brain Behav. 30, 230–248. 10.1159/000118648 3664264

[B53] Lim D. A. Huang Y. Alvarez-Buylla A. (2008). Adult subventricular zone and olfactory bulb neurogenesis, in Adult Neurogenesis, eds Gage F. H. Kempermann G. Song H. (New York, NY: Cold Spring Harbor Laboratory Press), 175–206.

[B54] Lindsey B. W. Tropepe V. (2006). A comparative framework for understanding the biological principles of adult neurogenesis. Prog. Neurobiol. 80, 281–307. 10.1016/j.pneurobio.2006.11.007 17218052

[B55] Ma D. Ming G. Gage F. H. Song H. (2008). Neurogenic niches in the adult mammalian brain, in Adult Neurogenesis, eds Gage F. H. Kempermann G. Song H. (New York, NY: Cold Spring Harbor Laboratory Press), 207–255.

[B56] Maler L. Sas E. Johnston S. Ellis W. (1991). An atlas of the brain of the electric fish *Apteronotus leptorhynchus*. J. Chem. Neuroanat. 4, 1–38. 10.1016/0891-0618(91)90030-G 2012682

[B57] Meek J. Nieuwenhuys R. (1998). Holosteans and teleosts, in The Central Nervous System of Vertebrates, eds Nieuwenhuys R. H. ten Donkelaar J. Nicholson C. (Berlin: Springer), 759–938.

[B58] Nottebohm F. (2002). Why are some neurons replaced in adult brain? J. Neurosci. 22, 624–628. 10.1016/so361-9230(02)00750-5 11826090 PMC6758507

[B59] Olivera-Pasilio V. Peterson D. A. Castelló M. E. (2014). Spatial distribution and cellular composition of adult brain proliferative zones in the teleost, *Gymnotus omarorum*. Front. Neuroanat. 8:83. 10.3389/fnana.2014.00088 25249943 PMC4157608

[B60] Olivera-Pasilio V. (2014). Distribución Espacial, Composición Celular y Capacidad Neurogénica de las Zonas Proliferativas del Cerebro de Gymnotus omarorum en la Vida Posnatal. Dissertation/masters'thesis, Universidad de la República, Montevideo.

[B61] Pellegrini E. Mouriec K. Anglade I. Menuet A. Le Page Y. Gueguen M.-M. (2007), Identification of aromatase-positive radial glial cells as progenitor cells in the ventricular layer of the forebrain in zebrafish. J. Comp. Neurol. 501, 150–167. 10.1002/cne.21222. 17206614

[B62] Radmilovich M. Barreiro I. Iribarne L. Grant K. Kirschbaum F. Castelló M. E. (2016). Post-hatching brain morphogenesis and cell proliferation in the pulse-type mormyrid *Mormyrus rume proboscirostris*. J. Physiol. Paris 110, 245–258. 10.1016/j.jphysparis.2016.11.007 27888101

[B63] Rahmann H. (1968). Autoradiographische Untersuchüngen zum DNs-Stoffwechsel (Mitose-Häufigkeit) im ZNS von Brachydanio rerio HAM: BUCH. (Cyprinidae, Pisces). J. Hinforsc. 10, 279–284. 5732947

[B64] Raymond P. Easter S. (1983). Postembryonic growth of the optic tectum in goldfish. I. Location of germinal cells and numbers of neurons produced. J. Neurosci. 5, 1077–1091. 10.1523/JNEUROSCI.03-05-01077.1983 PMC6564515 6842282

[B65] Richer-de-Forges M. Crampton W. G. R. Albert J. S. (2009). A new species of Gymnotus (Gymnotiformes, Gymnotidae) from Uruguay: description of a model species in neurophysiological research. Copeia 3, 538–544. 10.1643/CI-07-103

[B66] Richter W. Kranz D. (1970). Die Abhängigkeit der DNS-synthese in den matrixzonen des mesencephalons vom lebensalter der versuchstiere (*Lebistes reticulatus* – Teleostei): autoradiographische untersuchungen. Z Mikrosk Anat. Forsch 82, 76–92. 5480610

[B67] Rodríguez-Gómez F. J. Rendön-Unceta M. C. Sarasquete C. Mu-oz-Cueto J. A. (2000). Localization of tyrosine hydroxylase-immunoreactivity in the brain of the Senegalese sole, Solea senegalensis. J. Chem. Neuroanat. 19, 17–32. 10.1016/S0891-0618(00)00047-8 10882834

[B68] Rosillo J. C. Torres M. Olivera-Bravo S. Casanova G. García-Verdugo J. M. Fernández A. S. (2016). Telencephalic-olfactory bulb ventricle wall organization in *Austrolebias charrua*: cytoarchitecture, proliferation dynamics, neurogenesis and migration. Neuroscience 12, 63–80. 10.1016/j.neuroscience.2016.08.045 27593094

[B69] Sas E. Maler L. Tinner B. (1990). Catecholaminergic systems in the brain of a gymnotiform teleost fish: an immunohistochemical study. J. Comp. Neurol. 292, 127–162. 10.1002/cne.902920109 1968915

[B70] Sébert M. E. Weltzien F. A. Moisan C. Pasqualini C. Dufour S. (2008). Dopaminergic systems in the European eel: characterization, brain distribution, and potential role in migration and reproduction. Hydrobiología 602, 27–46. 10.1007/s10750-008-9288-1

[B71] Shumway C. A. (2008). Habitat complexity, brain, and behavior. Brain. Behav. Evol. 72, 123–*34*. 10.1159/000151472 18836258

[B72] Sullivan J. M. Benton J. L. Sandeman D. C. Beltz B. S. (2007). Adult neurogenesis: a common strategy across diverse species. J. Comp. Neurol. 500, 574–584. 10.1002/cne.21187 17120293 PMC1939924

[B73] Teles M. C. Sîrbulescu R. F. Wellbrock U. M. Oliveira R. F. Zupanc G. K. H. (2012). Adult neurogenesis in the brain of the Mozambique tilapia, *Oreochromis mossambicus*. J. Comp. Physiol. A Neuroethol. Sens. Neural. Behav. Physiol. 198, 427–449. 10.1007/s00359-012-0721-6 22491885

[B74] Terzibasi E. T. Baumgart M. Battistoni G. Cellerino A. (2012). Adult neurogenesis in the short-lived teleost *Nothobranchius furzeri*: localization of neurogenic niches, molecular characterization and effects of aging. Aging Cell 11, 241–251. 10.1111/j.1474-9726.2011.00781.x 22171971 PMC3437507

[B75] Vadodaria K. C. Gage F. H. (2014). SnapShot: adult hippocampal neurogenesis. Cell 156, 1114.e1. 10.1016/j.cell.2014.02.029 24581504

[B76] Wang C. Liu F. Liu Y. Y. Zhao C. H. You Y. Wang L.. (2011). Identification and characterization of neuroblasts in the subventricular zone and rostral migratory stream of the adult human brain. Cell Res. 21, 1534–1550. 10.1038/cr.2011.83 21577236 PMC3365638

[B77] Zupanc G. (2008). Adult neurogenesis in teleosts fish, in Adult Neurogenesis, eds Gage F. Kempermann G. Song H. (New York, NY: Cold Spring Harbor Laboratory Press), 571–592.

[B78] Zupanc G. Horschke I. (1995). Proliferation zones in the brain of adult gymnotiform fish: a quantitative mapping study. J. Comp. Neurol. 353, 213–233. 10.1002/cne.903530205 7745132

[B79] Zupanc G. K. H. (2006). Neurogenesis and neuronal regeneration in the adult fish brain. J. Comp. Physiol. A Neuroethol. Sens. Neural. Behav. Physiol. 192, 649–670. 10.1007/s00359-006-0104-y 16463148

[B80] Zupanc G. K. H. (2011). Adult neurogenesis in teleost fish, in Neurogenesis in the Adult Brain I, eds Seki T. Sawamoto K. Parent J. M. Alvarez-Buylla A. (Tokyo: Springer Japan), 137–168.

[B81] Zupanc G. K. H. Hinsch K. Gage F. H. (2005). Proliferation, migration, neuronal differentiation, and long-term survival of new cells in the adult zebrafish brain. J. Comp. Neurol. 488, 290–319. 10.1002/cne.20571 15952170

[B82] Zupanc G. K. Horschke I. Ott R. Rascher G. B. (1996). Postembryonic development of the cerebellum in gymnotiform fish. J. Comp. Neurol. 370, 443–464. 10.1002/(SICI)1096-9861(19960708)370:4<443::AID-CNE3>3.0.CO;2-4 8807447

