# Supplementary figures and images for: Cell Proliferation, Migration, and Neurogenesis in the Adult Brain of the Pulse Type Weakly Electric Fish, Gymnotus omarorum
Source: Front Neurosci. 2017 Aug 17;11:437. doi: 10.3389/fnins.2017.00437 (PMC5562682; doi:10.3389/fnins.2017.00437)

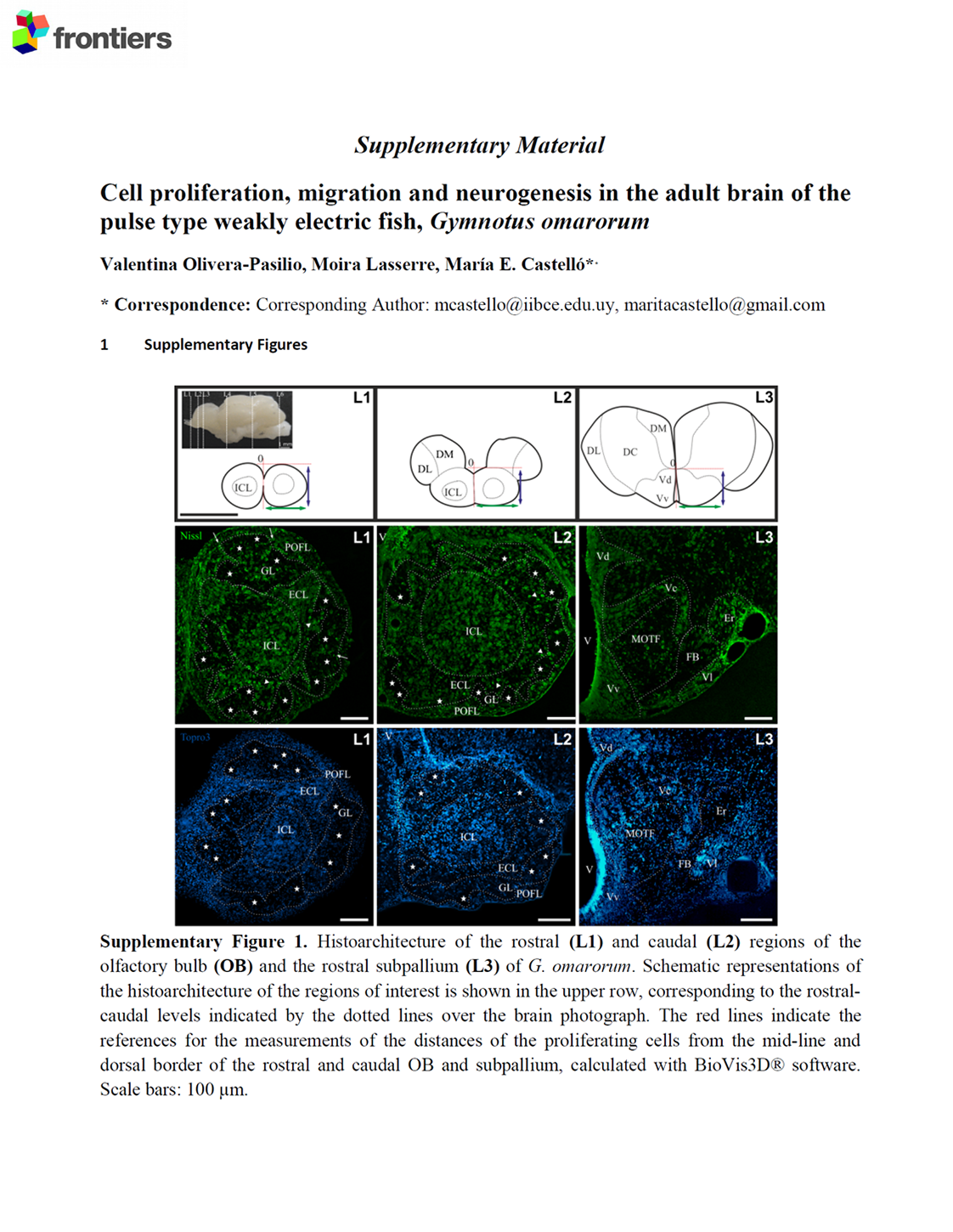

Supplement: Supplementary file 1 [file Image1.tif]

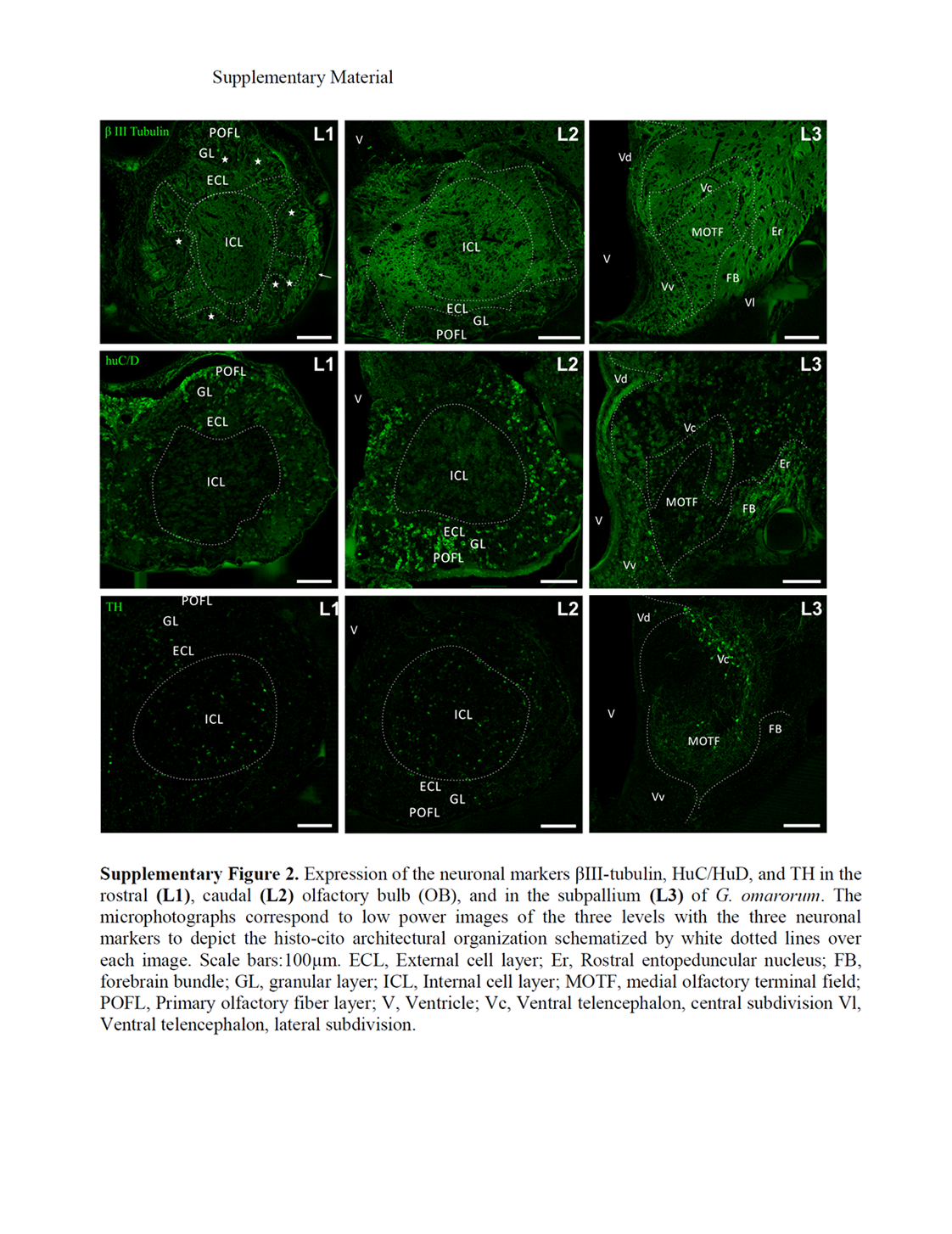

Supplement: Supplementary file 2 [file Image2.tif]

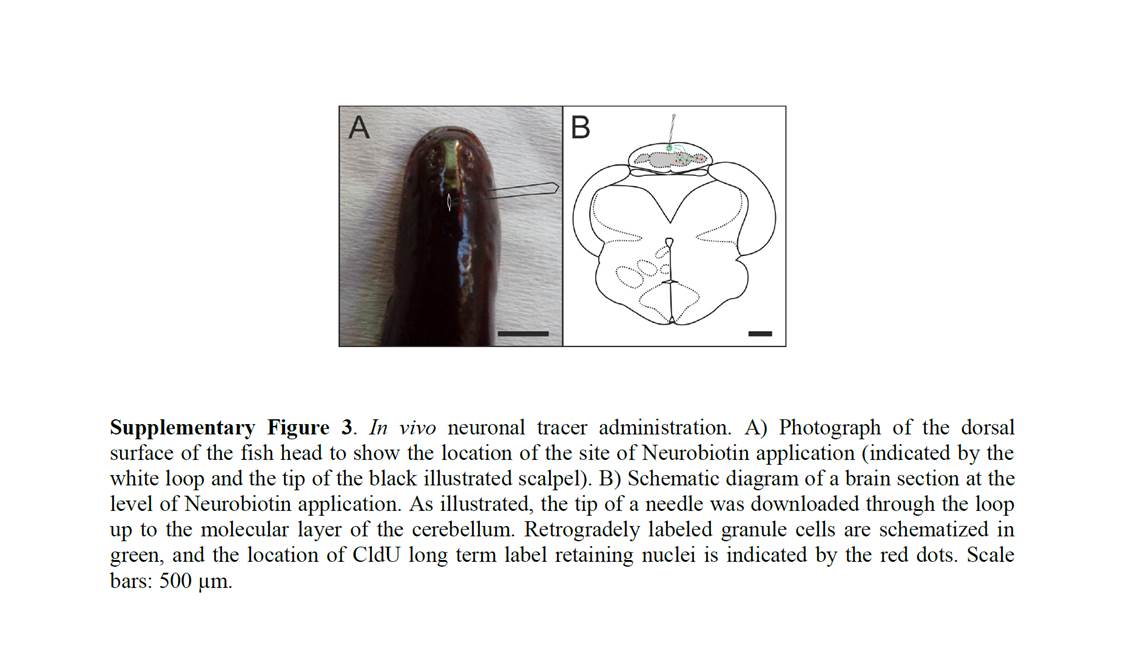

Supplement: Supplementary file 3 [file Image3.jpg]

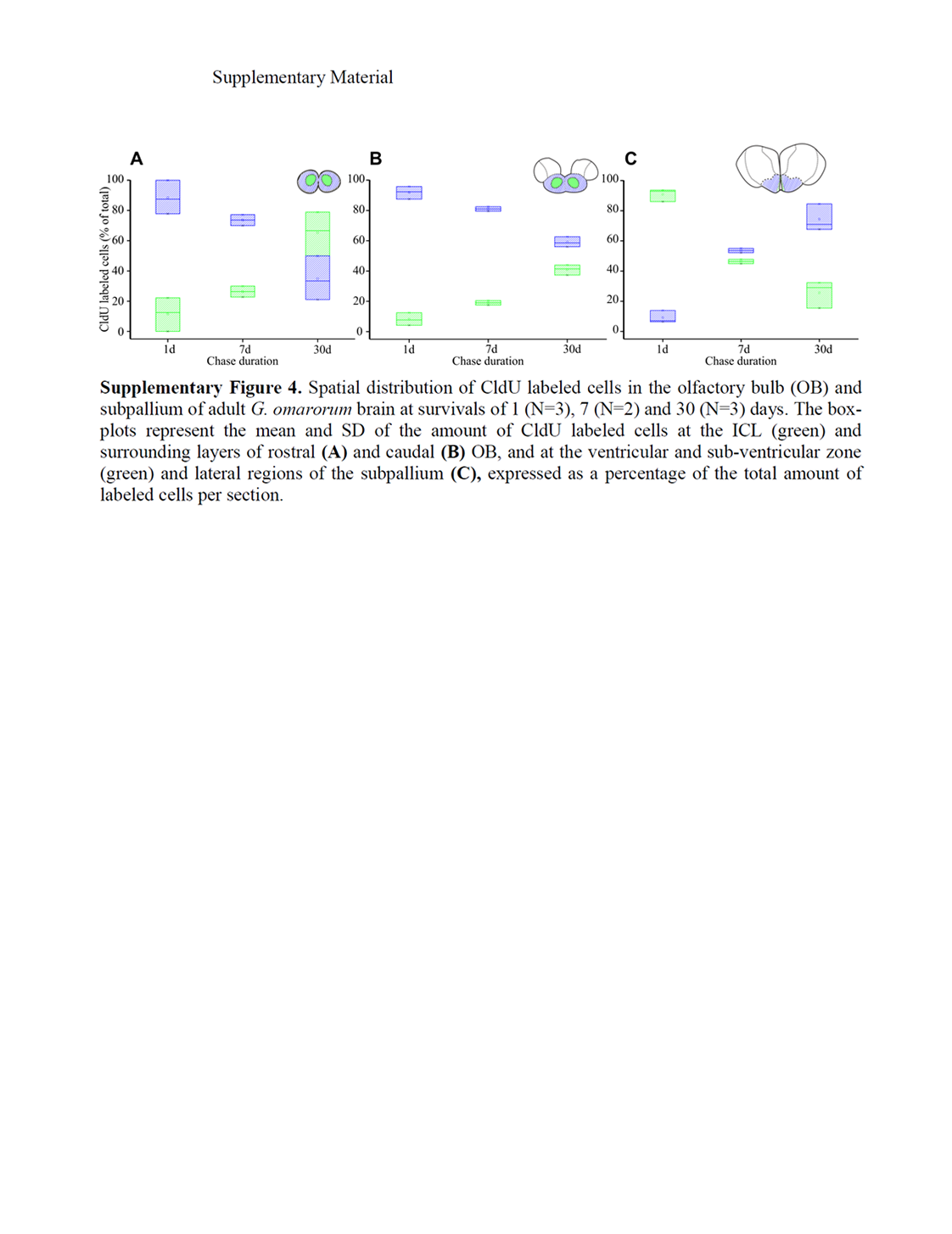

Supplement: Supplementary file 4 [file Image4.tif]

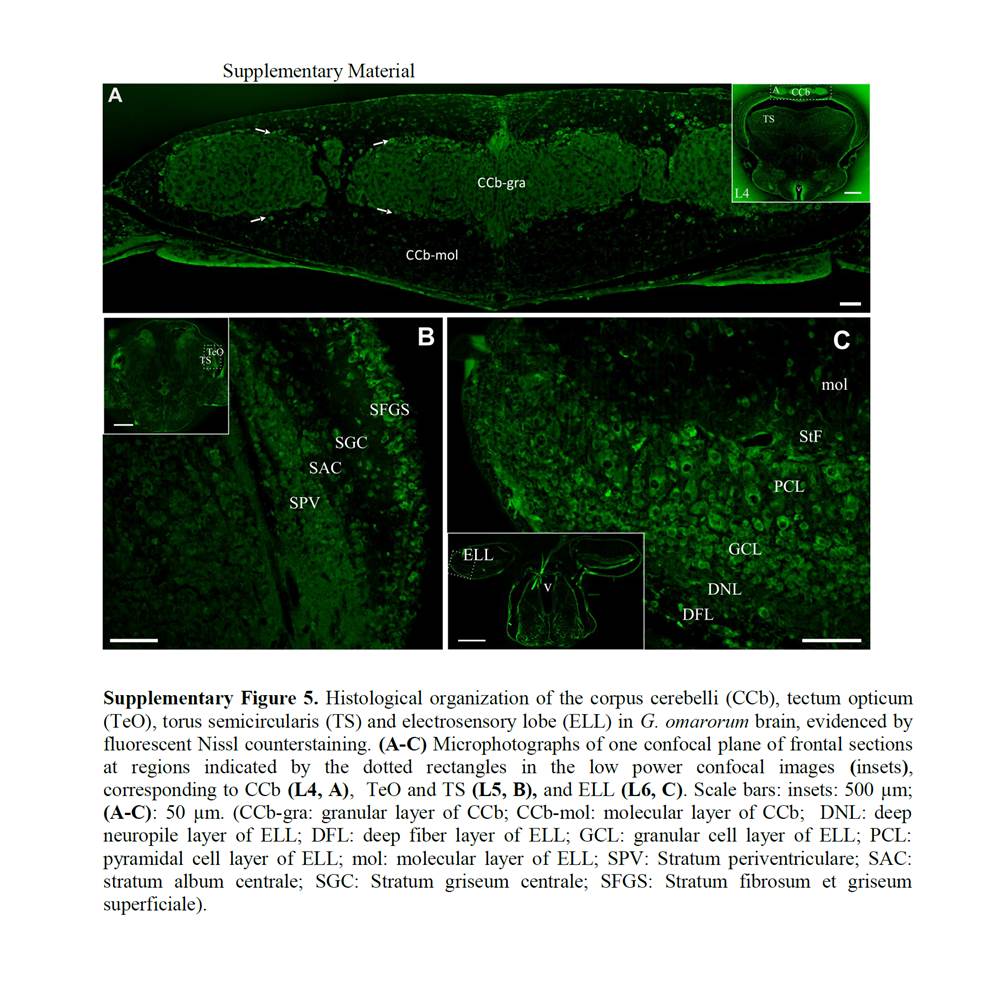

Supplement: Supplementary file 5 [file Image5.jpg]

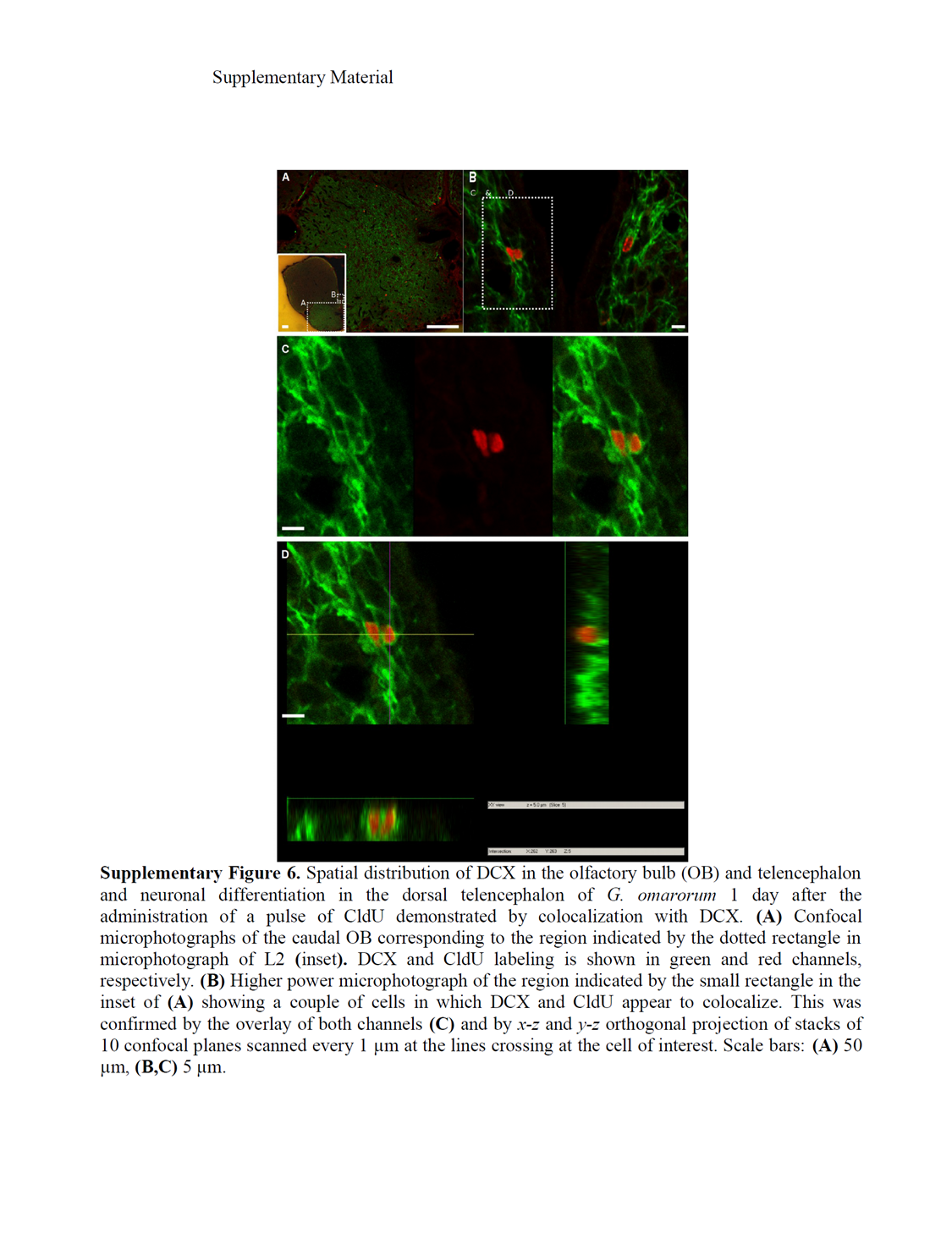

Supplement: Supplementary file 6 [file Image6.tif]

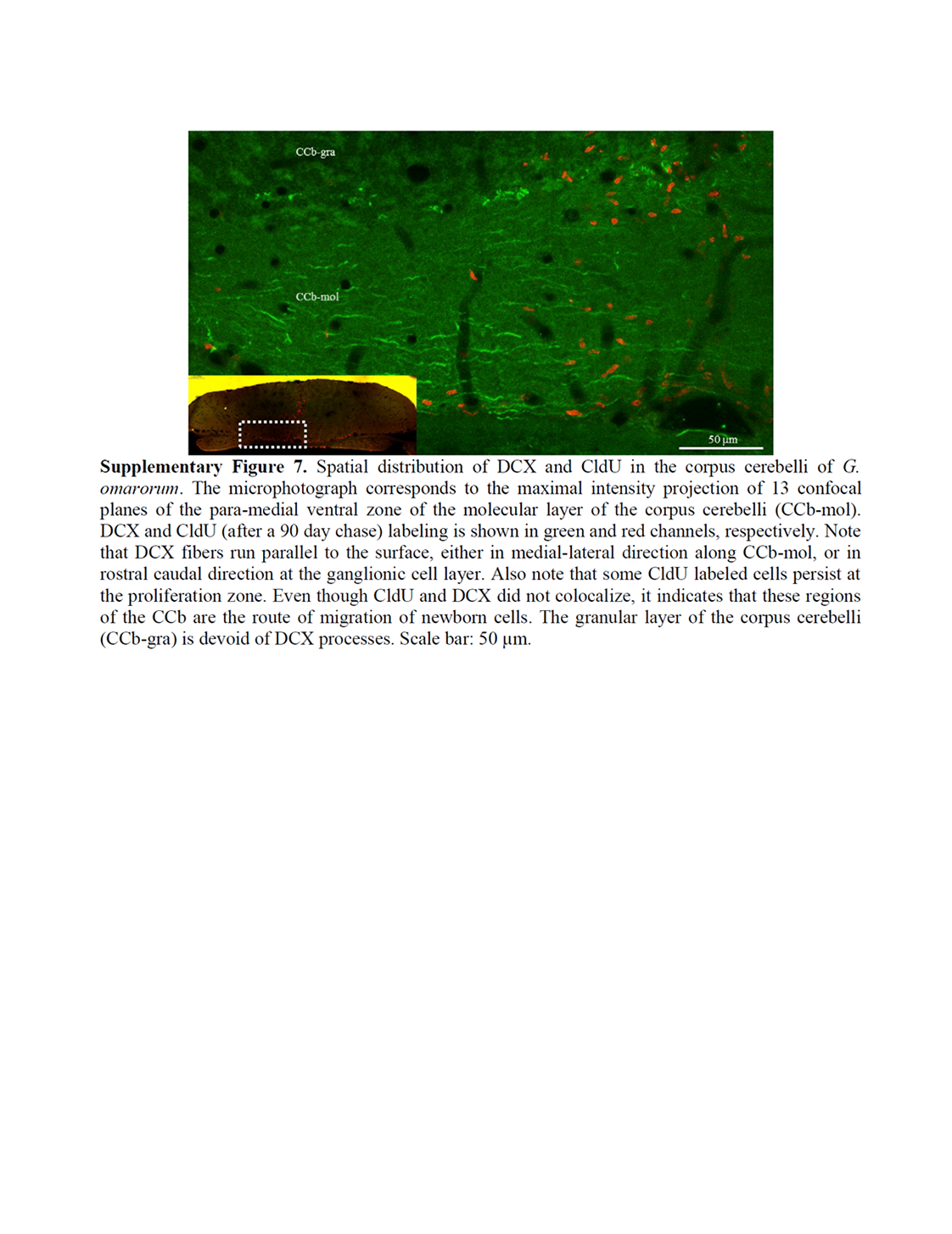

Supplement: Supplementary file 7 [file Image7.tif]

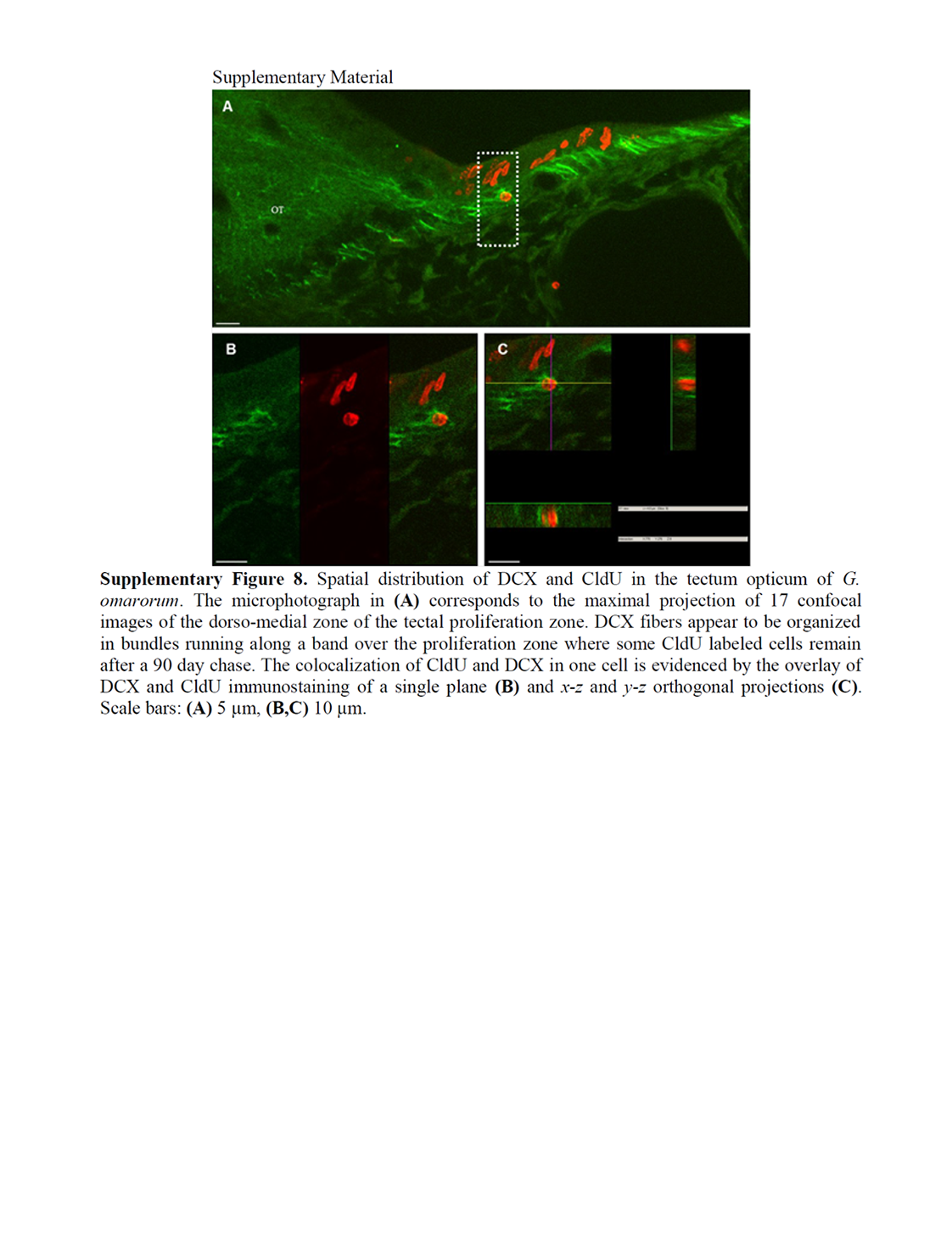

Supplement: Supplementary file 8 [file Image8.tif]
